# Pesticide safety behavior among vegetable farmers in Bangladesh: Evaluating the role of market aggregation services

**DOI:** 10.1016/j.heliyon.2024.e41013

**Published:** 2024-12-16

**Authors:** Ismat Ara Begum, Mohammad Jahangir Alam, Bhavani Shankar, Gregory Cooper, Karl Rich, Tamanna Mastura, Panam Parikh, Nazmun N. Ratna, Suneetha Kadiyala

**Affiliations:** a Department of Agricultural Economics, Bangladesh Agricultural University, Mymensingh, Bangladesh; b Department of Agribusiness and Marketing, Bangladesh Agricultural University, Mymensingh, Bangladesh; c Institute for Sustainable Food & Department of Geography, The University of Sheffield, Sheffield, United Kingdom; d Ferguson College of Agriculture, Oklahoma State University, USA; e Nutrition for Impact, 30 Cecil Street, Singapore, 049712, Singapore; f Faculty of Agribusiness & Commerce, Lincoln University, New Zealand; g London School of Hygiene and Tropical Medicine (LSHTM), London, United Kingdom

**Keywords:** Pesticides, LOOP aggregation, Vegetables, Bangladesh

## Abstract

Pesticide use in Bangladesh is disproportionately high in vegetable farming compared to other crops like cereals, pulses, and cash crops. This study delves into the knowledge, attitudes, and practices regarding pesticide use among vegetable farmers, focusing on the impact of a digital aggregation service implemented by Digital Green. Based on interviews with 120 vegetable farmers in the LOOP aggregation scheme and 120 non-LOOP vegetable farmers this study indicates that the farmers using the aggregation service have a moderately higher level of food safety knowledge. LOOP farmers scored higher in pesticide safety knowledge (67.83 %) compared to non-LOOP farmers (55 %). Regarding pesticide safety attitudes, LOOP farmers scored 17.39 %, while non-LOOP farmers 4.17 %, reflecting a generally poor attitude toward pesticide application. Regarding practices, 65.55 % of LOOP farmers adhered to scientifically sound methods, compared to 43.10 % of non-LOOP farmers. Although participation in the LOOP program significantly influenced farmers’ pesticide-related knowledge, attitudes, and practices, this study still identifies the need for targeted interventions and training to improve food safety practices among both groups.

## 1 Introduction

Bangladesh has become the world's third-largest vegetable producer, with an estimated production of 19.7 million metric tonnes (MT) in 2020–2021 [1]. This growth is driven by changing agricultural practices, as more farmers shift from traditional paddy cultivation to vegetable farming due to evolving climate patterns, including reduced rainfall and groundwater availability [2].

Pesticides play a critical role in maintaining high productivity in vegetable farming by protecting crops from pests and diseases [[3], [4], [5]]. Farmers often used to apply pesticides as a replacement for fertilizers in cultivating traditional and modern rice varieties, potatoes, spices, vegetables, and cotton [6]. This has led to an alarming increase in pesticide use, with over 37,000 MT applied in 2022 compared to 19,000 MT in 2000, driven by the substantial increase in vegetable production, which has led to a much higher use of pesticides in Bangladesh [7,8]. Depending on the pests that were encroaching on their crops, farmers primarily utilized Cypermethrin, Dichlorvos, Malathion, Carbofuran, Mancozeb, and Diazinon [9]. Also, this significant increase in pesticide usage is evidenced by the registration of 84 active chemicals with diverse formulations and 242 trade names for crop and vegetable protection [10].

According to the World Bank, there is a clear pattern of excessive pesticide usage among vegetable farmers in Bangladesh [11], as reported to apply pesticides to their crops 17–150 times over the growing season [9]. The amount of pesticide residue in vegetables grown in Bangladesh has been the subject of numerous research during the past few decades [[12], [13], [14], [15], [16], [17], [18]]. Furthermore, a significant number of pesticides utilized in Bangladesh were found in the list of banned or restricted substances (such as dichlorvos, dicrotophos, disulfoton, ethyl parathion, methyl parathion, mercury compounds, monocrotophos, phosphamidon) under international agreements such as the Stockholm Convention, the Basel Convention, the Rotterdam Convention [[19], [20], [21]]. This unbalanced application of pesticides has resulted in the presence of residues in vegetables, consequently contaminating fresh vegetables with potentially hazardous chemicals [22].

Pesticide residues directly or indirectly pass through the food chain and cause harm to humans and other organisms (aquatic or terrestrial). Insecticide poisoning affects 900 in every 100,000 people in Bangladesh [23]. According to the National Institute of Cancer Research and Hospital, about one-third of hospital patients who are found to have cancer every year are farmers [24]. Other pesticide-related health symptoms include illness, skin problems, neurological disturbances, and toxicity [25,26]. The significant escalation in both the use and subsequent consumption of pesticides has raised concerns regarding their potential ramifications on the health of farmers and the environment in Bangladesh [6,27,28].

Farmers can be exposed to pesticides during the storage, mixing, and application stages, making them more vulnerable to pesticide intoxication [29]. The health risk to farmers is even higher if there is poor knowledge or practices and an unfavorable attitude toward pesticide handling [30,31]. Available evidence on the knowledge, attitudes, and behaviour of smallholder farmers corroborates that unsafe pesticide usage is common in developing countries [[32], [33], [34], [35]]. Farmers in developing countries usually have little knowledge of the proper handling of pesticides, and do not normally handle the produce according to best agricultural practices [1,5], and are at a higher risk for health hazards [36] due to excessive exposure and inappropriate application procedures.

At the same time, vegetable farmers are unwilling to adopt adequate protective measures when using pesticides due to inadvertent habits and personal preferences [37]. Over 87 % openly admitted using little or no protective measures while applying pesticides [27]. Pursuing higher profits is the main barrier to adopting protective behaviors among Bangladeshi vegetable farmers [26]. This situation can be improved by education and training. However, according to a World Bank report, only 4 % of 820 Bangladeshi farmers surveyed had received formal training in pesticide use or handling. A similar lack of adoption of adequate protective measures by vegetable farmers when using pesticides has also been reported in China, despite farmers receiving adequate knowledge about pesticides [38]. This suggests that even extensive training may not change farmers' practices regarding the usage of pesticides [39] and that factors that influence farmers’ behaviors remain largely unknown [40].

Therefore, socio-demographic backgrounds such as age, sex, education, and income [35,37,[41], [42], [43], [44], [45], [46]] knowledge and experiences [41], inadequate knowledge and training regarding the safe use of pesticides, land ownership [11,42,43,[45], [46], [47], [48]], crop composition [11,44,46], and geographical location [11,44,46] are suggested to influence farmers’ safety-related behaviours [35,41].

However, market aggregation approaches can promote the sharing of best practices and resources, resulting in enhanced knowledge and adoption of proper food safety practices among participating farmers [49,50]. The aggregation system involves organizing and pooling individual quantities of agricultural products, which can lead to improvements in producer livelihoods while ensuring equitable distribution [50]. Farmers who participate in aggregation systems may become more aware of food safety regulations as a result of contact with aggregators, wholesalers, and customers [49,50]. For example, aggregators frequently ask farms to follow Good Agricultural Practices (GAP) and submit a food safety strategy [49]. The implementation of such a market aggregation scheme named LOOP aggregation service [51] in Bangladesh (2017-18) by Digital Green focuses on saving market transport costs and increasing market access for vegetable farmers in Jashore, implies a structured approach to agricultural practices and the farmers have the opportunity to acquire enhanced knowledge, share knowledge among the farming communities, develop a more positive attitude, and adopt safer practices compared to non-LOOP farmers. The LOOP program's digital approach to recording market transactions and providing farmers with real-time information may also influence their KAP, potentially improving their practices related to pesticide usage. LOOP farmers, operating within the structured framework of the aggregation system, may have different practices and knowledge levels than non-LOOP farmers who use traditional market supply methods. Therefore, this study found it interesting to compare the pesticide safety behavior (KAP) of two groups of farmers in Jashore, Bangladesh; one who participated in an aggregation service called LOOP, and the farmers who did not participate, coined as non-LOOP farmers. Comparing LOOP and non-LOOP farmers may provide us with an opportunity to assess the particular influence (if any) of such aggregation on pesticide KAP.

Given the increasing preference for horticulture, it is crucial to better understand the factors influencing farmers’ behaviors related to pesticide use for vegetable production. Thus, this study aims to explore the relationship between KAP and the socio-economic characteristics of vegetable farmers in Bangladesh.

This article is divided into four sections: Section 2 outlines the methods; Section 3 presents the results and their comprehensive discussion in Section 4; and Section 5 presents the conclusions and policy implications.

## 2 Methods

### 2.1 LOOP aggregation scheme and KAP of farmers

The "LOOP" aggregation scheme, implemented by Digital Green in April 2017, aims to improve transport logistics and increase market access for small-scale farmers in Bangladesh. According to Ref. [50], the LOOP scheme involves selecting local aggregators to collect produce from registered farmers within specific village clusters. These aggregators then transport the produce to designated markets, pooling the products from multiple farms to optimize transportation costs. By sharing transport expenses, farmers gain access to larger and more distant markets, potentially increasing their profit margins. Additionally, the LOOP system promotes more efficient and structured market transactions, allowing farmers to benefit from a more efficient approach to selling their vegetables. LOOP programs incentivized farmers to produce higher-quality vegetables and use fewer pesticides to receive higher premiums, reflected in better market prices compared to non-LOOP farmers′ produce. Interaction with peer farmers' and aggregators, along with market connections, may raise awareness of the economic and health benefits of using pesticides judiciously in vegetable production.

Knowledge, attitudes, and practices are considered fundamental factors within behavioral change models [40,52]. Knowledge can be defined as the cognitive comprehension of information, encompassing both conscious awareness and the non-symbolic interpretation of significance [53]. Attitudes are defined as a subjective appraisal, either positive or negative, of an objective entity [54]. Practice is a set of regular acts shaped by commonly held social norms and ideas [55]. In the context of pesticide use, farmers' behavior encompasses their understanding of pesticides (knowledge), their beliefs and perceptions about pesticide use and its consequences (attitudes), and the actual actions and methods employed in pesticide application and management (practices). Programs like the LOOP aggregation system, which includes farmer training or information-sharing components, are crucial in influencing several aspects of agricultural behavior. Farmers get knowledge on safe and responsible pesticide usage and build good attitudes towards implementing these practices through focused instruction. The LOOP program facilitates contacts among farmers, aggregators, and market stakeholders to share best practices, hence enhancing farmers' understanding and application of proper food safety measures.

### 2.2 Study area

**Fig. 1:**
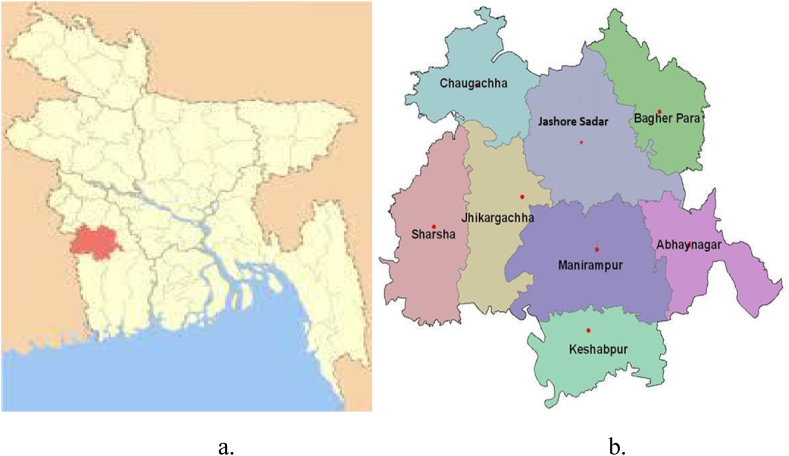
(a) Study area map of Jashore district (red); (b) Jashore District's Upazilas.

This study was conducted in Jashore Sadar, a sub-district within the Jashore district in northern Khulna, Bangladesh (Fig. 1a). Covering an area of 2607.2 sq. km, Jashore is a key agricultural region, with 81.69 % of households engaged in farming [56]. In 2017, approximately 14,105 ha of land in this district were dedicated to vegetable farming [57]. The Jashore district was selected for this study because Digital Green, an international non-government organization operating in Bangladesh and India, implemented the "LOOP" aggregation scheme in the Jashore district in April 2017. The scheme encompasses 30 villages across four unions - Churamankati, Haibatpur, Kashimpur, and Lebutala in Sadar upazila (Fig. 1b). The initiative aims to enhance transport logistics efficiency and boost profits for small-scale farmers.

### 2.3 Study design

This study assessed the impact of the LOOP aggregation scheme on KAP levels of a total of 240 vegetable farmers in Jashore Sadar using the farm-household survey data collected from July–August 2019. During the data collection phase, the study complied with all applicable ethical norms and principles. All of the vegetable farmers who took part in the study gave their informed consent, and their identity and confidentiality were carefully protected. This research analyzed two groups of farmers: (i) those who cultivated vegetables, sold independently, and were not engaged with the LOOP aggregation scheme in the non-LOOP villages (n = 120); and (ii) those who cultivated vegetables, sold, and were participants in the Loop aggregation scheme (n = 120).

A 'LOOP village' in this study denotes a community involved in Digital Green's LOOP aggregation scheme. Local farmers in these areas collaborate through the LOOP program to gather and collectively promote their vegetable produce. A local aggregator, mainly who has a personal transportation vehicle, manages the gathering and promotion of produce in a group of communities, promoting a more efficient and mutually beneficial method of agricultural marketing. The farmers were categorized into two groups based on their participation in the LOOP aggregation system to compare the knowledge, attitude, and practices (KAP) levels between these distinct farming communities.

LOOP farmers were chosen based on their registration in the LOOP aggregation system. A total of 120 farmers involved in the LOOP program were randomly selected from the 30 villages participating in the LOOP scheme. In contrast, 120 farmers from neighboring villages with similar socio-economic and geographic conditions, but not involved in the LOOP program, were randomly selected to form the non-LOOP group. These control villages were chosen to ensure comparability in land use, crop types, and market access between the non-LOOP and LOOP villages, with the only difference being the absence of LOOP participation. Random sampling was used in both groups to ensure that the sample accurately represented the population within each category.

This study hypothesizes that LOOP farmers will have higher KAP than non-LOOP farmers, and their preferences and practices are shaped by socio-economic factors such as sex, education, land ownership, and training [42,43,45,46,48] of two groups of farmers which will have an influence over their KAP levels about pesticide usage and these socio-economic factors are likely to have varied impacts. The authors acknowledge the potential for selection bias in this study, as LOOP villages may draw in farmers with particular characteristics like higher levels of education or land ownership. However, the authors also acknowledge that socio-economic characteristics such as sex, education, land ownership, and training could impact the preferences and practices of these groups. This research is based on cross-sectional data, which limits the capacity to establish causal relationships. It is assumed to have significant associations between participation in the LOOP program and KAP outcomes. Even though observable socio-economic characteristics were accounted for in the study, it is important to acknowledge the existence of unobserved variables or selection effects when interpreting the results. Hence, no claims of causation are made, and the findings are presented as associations with recognition of the inherent constraints of cross-sectional research.

### 2.4 Data collection and data analysis

The KAP of vegetable farmers was assessed both qualitatively and quantitatively using a pre-tested structured questionnaire. Data were collected through the *SurveyCTO* data-collection app, developed in consultation with the Digital Green team in Bangladesh. The ′Pesticides Safety: Knowledge, Attitudes and Practices′ was one of the modules in the master questionnaire. This module had 13 sub-sections covering knowledge, attitudes and practices, and socio-economic status. The knowledge subsection encompassed seven inquiries, and the attitude and practice subsections consisted of six questions, each on pesticide usage safety [26].

Descriptive and inferential statistics were used to assess LOOP vs non-LOOP farmers' safe pesticide practices and KAP levels. The descriptive statistical tools include mean, standard deviation, frequencies, and percentages to summarize and describe the key characteristics of the two groups of farmers (LOOP and non-LOOP). Then, using Bloom's original cutoff values [6], this study classified each item in the KAP variables into one of three quality levels: good (80%–100 %), average (60%–79 %), or poor (>60 %) [58]. Principal Component Analysis (PCA) was then employed to identify the main components (or principal components) that capture the majority of the variance in each KAP category and the scores for the latent variables (knowledge, attitudes, and practices) were calculated. Each KAP component (knowledge, attitudes, practices) consisted of multiple items/questions. Therefore, first, for each KAP component (knowledge, attitudes, practices), PCA identified a primary component that represented the main pattern of responses across all items. Then, this primary component was used to calculate a composite score for each KAP variable, effectively summarizing multiple items into a single score for knowledge, attitudes, and practices.

For inferential statistics, structural equation modeling (SEM) and path analysis were used to examine the causal relationships among the farmers’ behavior, focusing on KAP scores and socio-economic factors. Moreover, the Mann-Whitney test evaluated the knowledge, attitude, and practice scores of LOOP and non-LOOP farmers. The PCA, SEM, and path analysis were performed using the STATA 16 statistical software.

The use of SEM is appropriate for analyzing complex relationships between observed variables (such as socio-economic factors and KAP scores) and latent variables (for example, knowledge of farmers). SEM allows for simultaneous analysis of both direct and indirect effects which enhances the understanding of how socioeconomic factors influence KAP outcomes. Path analysis, a component of SEM was specifically used to model the direct relationship between socioeconomic factors (such as education, land ownership, training, experience, sex, and income) and farmers’ KAP results. Path analysis helps elucidate the strength and direction of these relationships while accounting for other factors in the model. The principal equation where ${KAP}_{i}$ represents the KAP score for the ith farmer, used for the path model is:

$${KAP}_{i}=\alpha +\beta_{1}X_{1}+\beta_{2}X_{2}+\beta_{3}X_{3}+\ldots +\beta_{n}X_{n}+\varepsilon_{i}$$

The study focuses on three main dependent variables, each corresponding to a component of the KAP framework. Each KAP component is assessed separately using its respective equation, allowing for examining the factors that directly influence knowledge, attitudes, and practices. The relationships between the KAP components and socio-economic factors are represented through the following regression equations, where each equation models one of the KAP components as the dependent variable, in relation to relevant independent variables. These independent variables include socioeconomic factors such as land ownership, training, level of education of the household head, LOOP participation, experience, knowledge, and harvesting time):

$$\text{Knowledge}:K_{i}=\alpha +\beta_{1}\left(Land\;Ownership\right)+\beta_{2}\left(Education\right)+\beta_{3}\left(LOOP\;Participation\right)+\beta_{4}\left(Experience\right)+\beta_{5}\left(Harvesting\;Time\right)+\varepsilon_{i}$$

$$\text{Attitudes}:A_{i}=\alpha +\beta_{1}(Training+\beta_{2}\left(LOOP\;Participation\right)+\beta_{3}\left(Knowledge\right)+\varepsilon_{i}$$

$$\text{Practices}:P_{i}=\alpha +\beta_{1}\left(Land\;Ownership\right)+\beta_{2}\left(Training\right)+\beta_{3}\left(LOOP\;Participation\right)+\beta_{4}\left(Experience\right)+\varepsilon_{i}$$

In the equations, $K_{i}$ represents the knowledge score of ith farmers; $A_{i}$ represents the attitudes score of ith farmers; $P_{i}$ represents the practices score of ith farmers; *α* is the intercept; *β1, β2, β3 …, βn* are the coefficients representing the effect of each independent variable of *X1, X2, X3 …, Xn* (such as land ownership, training, level of education of the household head, LOOP participation, experience, knowledge, and harvesting time) (Table 1) on the KAP scores; and $\varepsilon_{i}$ is the error term. The selection of independent variables for each equation is based on their expected relevance and theoretical justification regarding how each socio-economic factor might specifically impact Knowledge, Attitudes, or Practices. The Knowledge equation incorporates factors such as education and harvesting time, which are closely linked to acquiring and understanding information. The Attitudes equation highlights factors like training and LOOP participation, which shape perceptions and beliefs. Finally, the Practices equation includes variables such as experience and land ownership, which are more directly related to the practical application of knowledge in the field. This approach enables the study to address the unique drivers of each KAP component, offering a comprehensive understanding of the factors that influence farmers' knowledge, attitudes, and practices regarding pesticide safety.

**Table 1:** Definition of the dependent and independent variables.

Variables	Definition
**Dependent variables**	

Knowledge	Understanding of safe pesticide practices.
Attitudes	Beliefs and perceptions regarding the impact and necessity of safe pesticide use.
Practices	Actions or methods farmers employ when handling pesticides.

**Independent variables**	

Land Ownership	Farmers' status of land ownership (Yes = 1, No = 0).
Training	Whether the farmer received training related to pesticide safety (Yes = 1, No = 0).
Education Level	Years of education of the household head (number).
LOOP Participation	Participation in the LOOP aggregation scheme (Yes = 1, No = 0).
Experience	Years of farming experience (number).
Knowledge	Farmers' knowledge about pesticide safety (Yes = 1, No = 0)
Harvest Timing	Time to harvesting after pesticide application (number).

## 3 Results

### 3.1 Descriptive statistics

Pesticide use in Bangladesh was extremely high for vegetables compared to cereal crops, pulses, or cash crops (Table 2). Nearly all LOOP and non-LOOP farmers sprayed pesticides in their vegetable fields in the last growing season.

**Table 2:** Farmers most pesticide-sprayed crops.

Particulars	LOOP farmers N (%)	Non- LOOP farmers N (%)
**Crop category**		

Vegetables	118 (98.33)	120 (100)
Staple cereals (wheat, rice)	1 (0.83)	0 (0.00)
Pulses/millet/sorghum	1 (0.83)	0 (0.00)

**Characteristics**		

Spray routinely as per the crop calendar	50 (41.67)	14 (11.67)
Spray reactive based on pest observations	70 (58.33)	106 (88.33)

**Reasons for spraying pesticides**		

To control insects	104 (86.67)	104 (86.67)
To control disease	2 (1.67)	0 (0.00)
Other (i.e., growth hormone)	11.66	13.33

**Number of times sprayed.**		

0	1 (0.83)	0 (0.00)
1–10	32 (26.67)	14 (11.67)
11–20	36 (30.00)	42 (35.00)
21–30	16 (13.33)	14 (11.67)
31–40	10 (8.33)	15 (12.50)
41–50	11 (9.17)	9 (7.50)
Above 50	14 (11.67)	26 (21.67)

Around 42 % of LOOP farmers sprayed pesticides in their vegetable fields according to a schedule in the crop calendar, while the rest used pesticides in response to observed pest problems. In contrast, the majority (88.3 %) of the non-LOOP farmers used pesticides when pest problems emerged. Most farmers, both LOOP and non-LOOP, indicated that they used pesticides to control insects in their fields. A small percentage of LOOP (11.66 %) and non-LOOP (13.33 %) farmers also reported the use of pesticides as a growth hormone. LOOP farmers exhibit a uniform distribution across all spraying frequency intervals, whereas non-LOOP farmers tend to use pesticides more frequently, particularly in higher frequency ranges (Table 2).

The majority of farmers, both LOOP and non-LOOP, wore long-sleeved shirts (98.3 %), used a scarf/handkerchief to cover their face (over 80 %), and used a hat or other head covering (over 75 %) during pesticide application. However, only around 30 % of LOOP farmers and less than 10 % of non-LOOP farmers wore gloves or rubber boots during pesticide application, suggesting partial adoption of protective measures (Table 3).

**Table 3:** Protective measures followed by the LOOP and non-LOOP farmers.

Protective measures	LOOP farmers N (%)	Non- LOOP farmers N (%)
Long-sleeved shirt	118 (98.33)	118 (98.33)
Gloves	37 (30.83)	8 (6.67)
Hat or other head covering	93 (77.50)	91 (75.83)
Scarf/handkerchief to cover face	106 (88.33)	97 (80.83)
Rubber boots	34 (28.33)	11 (9.17)

A total of 67.8 % of LOOP farmers were knowledgeable about food-safety-related issues, especially about pesticide use and its impact on vegetable farming; this figure was only 55 % among non-LOOP farmers (Table 4). Results also found that 65.5 % of LOOP farmers practised protective measures related to pesticide use in vegetable farming, compared to 43.1 % of non-LOOP farmers. In contrast, the overall attitude towards food safety practices was poor. Only 17.39 % and 4.17 % of LOOP and non-LOOP farmers reported positive attitudes toward food safety practices.

**Table 4:** Key observations for KAP regarding pesticide safety.

Code	Questions on knowledge of food safety	%
K1	Which vegetable type did you spray the most with pesticides in the last year?	LP 67.83 % NL 55.00 %
K2	Which of the following best describes your approach to spraying/applying pesticides?	
K3	Typically, how many times in a growing season do you spray vegetables?	
K4	Who is the main person responsible for spraying/applying?	
K5	Does the sprayer read the contents of the pesticide bottle before spraying?	
K6	Do you agree that exposure to pesticides can have an adverse impact on human health?	
K7	How many days do you wait to harvest vegetables after spraying?	
	**Questions on attitude toward food safety**	

A1	Breathing in a pesticide	LP 17.39 % NL 4.17 %
A2	Being bitten by a mosquito	
A3	Getting pesticide on the skin	
A4	Swallowing pesticide	
A5	Consuming food from farms that have sprayed pesticides heavily	
A6	Pesticide containers can be reused safely after cleaning.	
	**Questions on practices related to food safety**	

P1	Long-sleeved shirt	LP 65.55 % NL 43.10 %
P2	Gloves	
P3	Hat or other head cover (e.g., *gamcha*)	
P4	Scarf/handkerchief to cover face	
P5	Rubber boots	
P6	Have you received training on the safe use of pesticides?	

**Note:** LP = LOOP group; NL = non-LOOP group.

LOOP farmers had significantly higher (*p* < 0.05) behavior scores, based on PCA, especially for knowledge and practices, than in the non-LOOP group. The findings also revealed that the scores for non-LOOP farmers’ behavior were negative for every aspect of KAP (Table 5).

**Table 5:** Evaluation of KAP among LOOP and non-LOOP vegetable farmers.

Farmers' behavior	Non-LOOP farmers		LOOP farmers		Mann–Whitney test
	Mean	SD	Mean	SD	*p*-value
Knowledge score	−0.0026	0.0127	0.0014	0.0124	0.0005∗∗∗
Attitudes score	−0.0033	0.0165	0.003	0.0244	0.7039
Practice score	−0.0112	0.0389	0.0048	0.0293	0.0002∗∗∗

### 3.2 Socio-economic factors and food safety behavior of farmers

Farmer participation in LOOP and their socio-economic characteristics influenced their level of knowledge regarding the safe use of pesticides (Appendix Figure 1). A positive relationship existed between knowledge and participation in the LOOP program (*p* < 0.10), status of land ownership (*p* < 0.05), level of education (*p* < 0.10), experience (*p* < 0.01), and vegetable harvesting times (*p* < 0.01) (Table 6). LOOP participation and socio-economic characteristics also influenced farmer attitudes towards the safe use of pesticides and vegetable food safety. Path analysis of farmer attitudes regarding vegetable food safety is presented in Appendix Figure 2. Farmer attitudes were positively influenced by training facilities (*p* < 0.05), LOOP status (*p* < 0.05), and farmer knowledge (*p* < 0.01) (Table 6). A similar positive association was also observed between farmer participation in the LOOP aggregation scheme, their socio-economic characteristics, and farmers’ practices regarding the safe use of pesticides and vegetable food safety (Appendix Figure 3). Land ownership (*p* < 0.10), training status (*p* < 0.01), LOOP status (*p* < 0.01), and farmer experience (*p* < 0.01) positively influenced farmer practices (Table 6). The model fitness indicators verify the fitness of the path analysis for KAP regarding vegetable food safety, and it finds that the model fits well (Appendix Table 1) [59,60].

**Table 6:** Associations among socio-economic factors and knowledge, attitudes, and practices.

Variables	Knowledge		Attitudes		Practices	
	Coefficient	St. err.	Coefficient	St. err.	Coefficient	St. err.
Land ownership (yes = 1)	0.0037∗∗	0.0016	–	–	−0.00773∗	0.0041
Training (yes = 1)	–	–	0.0047∗∗	0.0025	0.0307∗∗∗	0.0036
Level of education of household head (years)	0.0018∗	0.0010	–	–	–	–
LOOP participation (yes = 1)	0.0026∗	0.0014	0.0053∗∗	0.0026	0.0170∗∗∗	0.0038
Farmer's experience (yes = 1)	0.0093∗∗∗	0.0024	–	–	0. 0232∗∗∗	0.0062
Farmers' knowledge (yes = 1)	–	–	0.0108∗∗∗	0.0027	–	–
Harvesting time (number)	0.0019∗∗∗	0.0003	–	–	–	–
Constants	−0.0114	0. 0020	−0.0140	0.0052	−0.0524	0.0073

**Notes:** ∗∗∗, ∗∗, and ∗ indicate the level of significance at 1 %, 5 % and 10 %, respectively. Only reported the significant variables.

## 4 Discussion

This study investigated the KAP of vegetable farmers concerning safe pesticide usage by interviewing 120 LOOP farmers and 120 non-LOOP farmers in Bangladesh. It revealed a significant difference in practices among LOOP and non-LOOP farmers. After controlling for factors such as education and land ownership that may be correlated with both LOOP participation as well as KAP outcomes, this study can confirm that LOOP participation is positively associated with farmer KAP regarding pesticide use and vegetable food safety, highlighting the prospective advantage of participating in the LOOP program.

Pesticides are crucial for increasing agricultural productivity and protecting crops against pest infestations [61,62]. However, the application of pesticides must be managed carefully based on the observations of pest-related issues. In Bangladesh, a significant proportion of farmers lack appropriate knowledge or exhibit indifference toward the optimal timing and frequency of pesticide application during vegetable cultivation [3,26]. It is, therefore, imperative to gain insight into the extent of farmer knowledge and adherence to safe pesticide usage to develop effective educational and policy approaches that mitigate the adverse health and environmental effects of pesticide use.

Previous studies have reported a significant positive association between KAP levels and pesticide use behaviors among farmers [63,64] which is further reiterated by the results of the current study. The findings of this research demonstrate a multifaceted correlation between farmers' attitudes toward food safety and their participation in the LOOP program, exposure to training, and level of knowledge. Farmers in the LOOP program status exhibited significantly more favorable attitudes than those who did not have this opportunity. This suggests that specialized initiatives that offer knowledge-sharing opportunities, like the LOOP program, could enhance individual inclinations toward adhering to food safety practices. This is further reinforced by the positive associations between active participation in the LOOP program, the availability of training facilities, and a higher level of knowledge.

The findings are consistent with other research [35,47,63,65,66], which suggests that the provision of training to farmers had a substantial impact on shaping their attitudes. The improved attitudes observed may be attributed to the enhanced knowledge about pesticide safety [38,47,67]. Nevertheless, it is crucial to interpret these findings with caution, as a generally low percentage of farmers exhibited a positive attitude towards food safety, regardless of their participation in the LOOP program.

Interestingly, despite poor attitudes towards pesticide usage, farmers in the LOOP program generally demonstrated good practices. They followed a systematic approach to the application of pesticides and adhered to precautionary measures, consistent with findings by Refs. [68,69], highlighting the beneficial role of initiatives like LOOP.

In addition to initiatives such as LOOP, socio-economic factors such as land ownership and farming experience also significantly influence farmers' willingness to adopt safe practices [37,[41], [42], [43],^45^,^47^,^48^]. Farmers with more extensive farming experience and smaller land holdings are more likely to prioritize safe practices [70,71], as those with smaller land holdings may better understand the significance of employing safe practices to maximize the utilization of limited resources [72]. The influence of farming experience implies that longer involvement in agriculture equips farmers with knowledge and skills that make them more receptive to adopting safe practices [73]. In this study, the comprehensive results indicated a positive correlation between the socioeconomic status of vegetable farmers and their KAP concerning pesticide safety. This underscores the critical influence of socio-economic factors on the development of food safety behaviors among vegetable farmers. The authors acknowledge the significant impact that increased levels of socioeconomic status, such as education and land ownership, can have on knowledge outcomes. This study considers and adjusts for these factors when assessing the impact of participation in the LOOP programme. Farmers with greater socio-economic status are found more inclined to participate in the LOOP programme. Even after accounting for these factors, participation in the LOOP programme remains positively associated with improved knowledge, attitudes, and practice outcomes. This suggests that the LOOP programme positively influences food safety behaviours regardless of socio-economic level. However, to address potential socio-economic biases, targeted training initiatives are essential to fill knowledge gaps and enhance food safety procedures, especially for farmers with lower socio-economic status.

This research holds significant implications for policymakers and agricultural development organizations. It underscores the potential impact of implementing focused interventions and educational initiatives to address knowledge disparities and promote safer pesticide application practices across the agricultural community. The positive influence of the LOOP program on farmer KAP demonstrates the potential for similar initiatives incorporating elements that enhance farmer knowledge, attitudes, and practices related to pesticide safety to promote safer and more sustainable agricultural practices. Thus, the recommendations include expanding and improving programs such as LOOP, evaluating the programs based on participant feedback for improvement, emphasizing improving farmer education and awareness, increasing access to protective gear, promoting more responsible pesticide use practices, and multi-stakeholder approaches to ensure a comprehensive effort. Additionally, efforts should be made to tailor interventions to the distinct socioeconomic contexts of various agricultural communities to maximize their effectiveness and sustainability.

However, the authors acknowledge certain limitations of this research, including its cross-sectional design, which presents a snapshot of farmer practices at a particular time and limits causal inference. Longitudinal studies capturing temporal dynamics to help researchers better understand how knowledge, attitudes, and practices develop over time are warranted to develop robust strategies. The effect of contextual factors cannot be ignored, and a replication of similar studies in other parts of Bangladesh is recommended to get more comprehensive country-specific insights on food safety practices among vegetable farmers.

## 5 Conclusion

The lack of safety measures and established practices for pesticide use among vegetable farmers poses serious threats to food safety in Bangladesh. Our findings emphasize the need to improve farmer attitudes and practices regarding pesticide use to enhance vegetable food safety in Bangladesh. To improve food safety in vegetable farming, it is imperative to adopt a comprehensive strategy that incorporates cooperative efforts, focused educational programs, and the recognition of socio-economic factors to foster enduring and beneficial shifts in farmer behaviours. The strategies like LOOP scheme with tailored training, and awareness campaigns may offer an effective pathway to bridge the knowledge gap, create positive attitudes, and promote responsible pesticide use practices among vegetable farmers. Additionally, participatory training modules should be developed to address specific pesticide-related challenges faced by farmers. These modules should emphasize safe handling, application techniques, and awareness about the appropriate waiting periods before harvesting vegetables. The government and related authorities can take significant strides towards ensuring safer agricultural practices and safeguarding the health of both farmers and consumers through effective policy measures.

## CRediT authorship contribution statement

**Ismat Ara Begum:** Writing – original draft, Methodology, Formal analysis, Data curation, Conceptualization. **Mohammad Jahangir Alam:** Writing – review & editing, Visualization, Validation, Supervision, Software, Methodology, Funding acquisition, Conceptualization. **Bhavani Shankar:** Writing – review & editing, Validation, Supervision, Resources, Project administration, Methodology, Conceptualization. **Gregory Cooper:** Writing – review & editing, Visualization, Validation, Data curation, Conceptualization. **Karl Rich:** Writing – review & editing, Validation, Supervision, Resources, Methodology, Conceptualization. **Tamanna Mastura:** Writing – review & editing, Software, Investigation, Formal analysis, Data curation. **Panam Parikh:** Writing – review & editing, Validation, Supervision, Formal analysis, Data curation. **Nazmun N. Ratna:** Writing – review & editing, Methodology, Investigation, Conceptualization. **Suneetha Kadiyala:** Writing – review & editing, Validation, Project administration, Funding acquisition, Conceptualization.

## Data and code availability statement

Data and code will be made available on request.

## Financial disclosure

"The authors gratefully acknowledge financial support from the Market Intervention for Nutritional Improvement (MINI) project funded by the Bill & Melinda Gates Foundation (BMGF) and the UK Government's Foreign, Commonwealth and Development Office (FCDO) (Grant No. OPP1182694). The views expressed in this work are those of the creators and do not necessarily represent those of BMGF, FCDO, or Digital Green."

## Ethical statement

This study adhered to the guidelines of Lincoln University New Zealand and obtained ethical approval from the Lincoln University Human Ethics Committee (No. 2019-38; Dated July 11, 2019). Written informed consent was obtained from all the participants. Those who voluntarily chose to participate were required to review and sign a consent form indicating their informed consent to participate in the study. The questionnaires were anonymized, and respondents were free to opt out of participation in the survey. The participants were informed that the data would be kept anonymous and confidential. The data will only be used for academic and non-commercial purposes.

## Declaration of competing interest

The authors declare that they have no known competing financial interests or personal relationships that could have appeared to influence the work reported in this paper.
