# Supplementary material for: Pesticide safety behavior among vegetable farmers in Bangladesh: Evaluating the role of market aggregation services
Source: Heliyon. 2024 Dec 16;11(1):e41013. doi: 10.1016/j.heliyon.2024.e41013 (PMC11721242; doi:10.1016/j.heliyon.2024.e41013)
Supplement: Multimedia component 1 [file mmc1.docx]

**MINI household (LOOP participant) survey**

**For enumerator:**

**Definitions**

*Primary respondents:* There are two parts to this survey. The first part from section B to section L should be answered by a primary respondent. Primary respondents are those which are self-identified as the primary members responsible for decision making, both social and economic, within the household. They are usually household head or spouse, however, the respondent can also be another member as long as there are one male and one female aged 18 and over. They must be especially knowledgeable about agricultural aspects. The second part, composed of sections M and N, should be answered by an adult female respondent, preferably also a household head or spouse.

*Reference period.* The reference period that will be used for the survey is the ‘last twelve months’ (covering the periods **Kharif-1 (16 March-15 July 2018), Kharif-2 (16 July-15 November 2018) and Rabi (16 November 2018-15 March, 19).** We will refer to most questions regarding agricultural and income generating activities to this period. Sometime we will instead refer to the preceding 12 months before the survey – it will be made clear where this is the case. We also sometimes use recall questions on the situation five years ago. While it may be difficult for the household to exactly remembers the situation then, we are interested in approximate evaluations for that period.

***Household.*** A household is a group of people who live together and eat together. In our survey, a household member is someone who has lived in the household at least 6 months and at least half of the week in each week in those months. Even those persons who are not blood relations (such as servants, lodgers, or agricultural labourers) are members of the household if they have stayed in the household at least 3 months of the past 6 months and take food together. Generally, if one person stays more than 3 months out of the last 6 months outside the household, they are not considered household members. We do not include them even if other household members consider them as household members.

***Leasing.*** A plot is considered “leased-out” when somebody else (other than the farmer interviewed) pays a fixed amount of money to the farmer to rent that plot for a fixed period of time and gets in exchange the produce of that plot. A plot is “leased-in” when the interviewed farmer leases the land from another person and uses it and keeps (for his own use or sale) the produce from that plot. A sharecropping arrangement is such an arrangement where part of the produce on the field goes to the owner and part of it goes to the person who cultivates (“sharecrops-in”) the land.

Question number format

e.g. A101 A = module code; 1 = sub-module code; 01 question number in sub-module

Before beginning the interview, it is necessary to introduce both participants to the survey and obtain their consent to participate (i.e. the head male and head female household member). Please read the survey information sheet provided and make it clear to them that their participation in the survey is voluntary. Once agreed, you can swipe forward to continue.

Household member availability:

Is the household member available? _____________________Yes________________No

Is it possible to question the head male household members during this survey? _________________Yes_______No

Is it possible to question another male adult during this survey? ________________________Yes_____________No

Is it possible to question the head female household members during this survey? ___________Yes____________No

Is it possible to question another female adult during this survey? ______________________Yes________________No

If a female is not available, Please stop the survey

Consent:

Does the male household member consent to participate in this form? _____________Yes____________No

Does the female member consent to participate in this form? __________________Yes_______________No

As both participants have not given their consent, PLEASE STOP this survey and make a note of the consent refusal and Confirm which household member is primarily responsible for the household's agricultural activities

# Module A: Household identification

| **Household identification** | **Code** | **Interview details** | **Code** | **Interview details** |  |
| --- | --- | --- | --- | --- | --- |
| A101: Household identification | \|  \|  \| \| --- \| --- \| | A104: Start and end time of interview (hh:mm) | \|  \|  \| : \|  \|  \| \| --- \| --- \| --- \| --- \| --- \| | A106: GPS Coordinates (decimal degrees) | Lat ____  Long ____ |
| A102: Union | \|  \|  \| \| --- \| --- \| | A105: Date of interview (dd/mm/yyyy) | \|  \|  \| / \|  \|  \| / \|  \| \| --- \| --- \| --- \| --- \| --- \| --- \| --- \| | A107: LOOP household (N=0, Y=1) | _________ |
| A103: Village | \|  \|  \| \| --- \| --- \| |  |  |  |  |

| **Household Identification code** | **Union code** | **Village code** | |
| --- | --- | --- | --- |
| Head male………………………..1  Head female……………………...2  Another adult male…………………3  Another adult female……………….4 | Haibatpur…………….1  Lebutala………………2  Kashempur……………3  Churamonkathi…………4 | \| Choto Haibatpur..................1 \| \| --- \| \| Mothurapur…………………2 \| \| Uttar Lolitadaha……………..3 \| \| Boro Haibatpur……………..4 \| \| Mire laukhali………………..5 \| \| Andalpota…………………..6 \| \| Kashempur………………….7 \| \| Noudaga…………………….8 \| \| Hapani……………………….9 \| \| Dokkin Lolitadah……………10 \| \| Tirerhat……………………….11 \| \| Rahamatpur………………….12 \| \| Natuapara…………………….13 \| \| Doherpara……………………14  Bijoynagar…………………….15 \| | \| Ghona………………………….6 \| \| --- \| \| Anayetpur…………………….17 \| \| Nischintopur………………….18 \| \| Somaspur……………………19 \| \| Baliaghat……………………..20 \| \| Dalennagar………………….21 \| \| Cowdia………………………22 \| \| Muradgor……………………23 \| \| Kadirpara……………………24 \| \| Lebutala…………………….25 \| \| Vogolpur……………………26 \| \| Dakatia……………………..27 \| \| Birnarayanpur……………..28 \| \| Kissmat…………………….29 \| \| Komlapur…………………..30  Others...............................31 \| |

# Module B: Household characteristics and demographics

**Enumerator: The questions in the below table apply to all household members (see the first page for a definition of household members). Please start with the household head (i.e. HH member #1)**

**Respondent ID:** Household head.

***Enumerator read:*** We would like to start by asking you questions to introduce yourself, your living arrangements and employment status. The answers to these questions will remain anonymous to people outside of the research team.

How many persons live in this household? (Please include the respondent in the answer)___________________

**Household characteristic**

| HH Member code | Relation with the household head | Gender | Age | Highest education attained | In school (studying) | Current main occupation | Location of employment |
| --- | --- | --- | --- | --- | --- | --- | --- |
|  | **Code 1** | *1. Male*  *2. Female* | Years | **Code 2** | *1. Yes*  *2. No* | **Code 3** | **Code 4** |
|  | B101 | B102 | B103 | B104 | B105 | B106 | B107 |
| 1 |  |  |  |  |  |  |  |
| 2 |  |  |  |  |  |  |  |
| 3 |  |  |  |  |  |  |  |
| 4 |  |  |  |  |  |  |  |
| 5 |  |  |  |  |  |  |  |
| 6 |  |  |  |  |  |  |  |
| 7 |  |  |  |  |  |  |  |
| 8 |  |  |  |  |  |  |  |
| 9 |  |  |  |  |  |  |  |
| 10 |  |  |  |  |  |  |  |

**Enumerator: the below questions are only about the household head.**

B108: Does the head of the household hold a (No = 0; Yes = 1):

(a) Vulnerable Group Feeding (VGF) card ________

(b) Vulnerable Group Development (VGD) card ________

(c) Widow/Old age allowances:

(d) Maternity allowance/lactating allowances: ________

B109: Head of household’s religion: ________ (1.Muslim; 2. Hindu; 3. Christian; 4. Buddhist; 5. Other)

B110: Family status of Household head___________________ (1. Single/Unmarried, 2. Married, 3. Divorced, 4. Widowed, 5. Other)

**Codes for Module B: Household characteristics and demographics**

| **CODE 1** (B101) Relationship with household head | **CODE 2** (B104) Education level/year of schooling | **CODE 3** (B106) Current main occupation | **CODE 4** (B107) Location of employment |
| --- | --- | --- | --- |
| Household head…....……..1  Spouse……………………..2  Son/daughter………..……..3  Son/daughter-in-law………4  Grandson/granddaughter..5  Mother/Father.…………….6  Brother/sister………………7 Nephew/niece……………..8  Nephew/niece of spouse…..9  Cousin of household head.10  Brother/sister-in-law……….11  Mother/father-in-law……....12  Cousin of household head’s spouse…13  Another relative……………….14  Maid………………………….15  Unsure ………………………99 | Not literate………………..1  Literate without formal schooling………………2  Literate but below primary (less than grade 1)…..3  Primary (1-5)……………..4  Middle (6-8)………………5  Secondary (8-10)………..6  Higher secondary (11-12)……………………..7  Diploma/certificate course………………...8  Graduate………………...9  Masters//above ….10  Vocational training ............11  Unsure …………………99 | Day labourer………1  Salaried worker……..2  Self-employed………3  Farming……………...4  Trader………………..5  Student………………6  Housewife…………...7  Retired……………....8  Unemployed………...9  Physically handicapped........10  Other (specify)……..11  Unsure.……………99 | This village…………….1  Another village in this district …2  This district urban ……3  Another district rural ………….4  Another district urban ……...5  Unsure ……………….99 |

# Module C: Land ownership

**Enumerator read:** Now I am going to ask you a series of questions about the land your household lives on, uses for productive purposes-- that is leases or sharecrops, owns or rents out. I would like you to consider not only the land your household uses now but also the land your household may have used during the past year. Please start with the largest piece of land and move to the smallest piece of land in terms of area. By a piece of land, I mean one continuous piece, which is used predominantly for the same purpose and managed by the same person or group of people. Include the homestead and home garden production.

**Respondent ID: ______ (from Module B)**

| Land ID | What is the area of [LAND] now? | | What is the ownership status of the [LAND] now?  **CODE 2** | What is the tenancy status of now [LAND]?  **CODE 3** | If you were to buy a similar plot of [LAND] today, how much would you pay (counting all improvements)?  (Tk) | What was the main use of [LAND] during the past year?  If **1 (>> continue)**  If **2 – 7 (>> next piece of land)**  **CODE 4** | How would you rate the overall quality of this [LAND]?  CODE 5 | What is the irrigation status of this [LAND]?  **CODE 6** | Land level  *1. Plains which can be flooded*  *2. Plains not flooded*  *3. High lands*  Code above | What were the three major crops in terms of the area allocated on [LAND] during the past year (Kharif-1 (16 March-15 July), 2018, Kharif-2 (16 July-15 November), 2018 and Rabi (16 November-15 March), 2018-19  *[List up to three with primary crop first]*  **CODE 7** | | |
| --- | --- | --- | --- | --- | --- | --- | --- | --- | --- | --- | --- | --- |
|  | a. Unit  **CODE 1** | b. Area |  |  |  |  |  |  |  |  |  |  |
| **ID** | C101a | C101b | C102 | C103 | C104 | C105 | C106 | C107 | C108 | C109a | C109b | C109c |
| 01 |  |  |  |  |  |  |  |  |  |  |  |  |
| 02 |  |  |  |  |  |  |  |  |  |  |  |  |
| 03 |  |  |  |  |  |  |  |  |  |  |  |  |
| 04 |  |  |  |  |  |  |  |  |  |  |  |  |
| 05 |  |  |  |  |  |  |  |  |  |  |  |  |
| 06 |  |  |  |  |  |  |  |  |  |  |  |  |
| 07 |  |  |  |  |  |  |  |  |  |  |  |  |
| 08 |  |  |  |  |  |  |  |  |  |  |  |  |

C110: What was the total area of the agricultural land available to you 2 years ago? ________ (number) _______ (unit – CODE 1)

**Codes for Module C**

| **CODE 1 (C101a): Area unit** | **CODE 2 (C102): Land Ownership** | **CODE 3 (C103): Tenure Status** | **CODE 4 (C105): Use status** | **CODE 5 (C107): Land quality** | **CODE 6 (C108): Irrigation method** | **Code 7 (C109) Crop types** |
| --- | --- | --- | --- | --- | --- | --- |
| Hectares…………….1  Square meters……...2  Acres…………………3  Feet…………………..4  Bigha…………………5  Kattha………………..6  Decimal ....................7  Unsure ……………...99 | Government/Khash land……………….1  Leasehold ……,…2  Rented -------------3  Mortgaged-----------4  Other (specify)…..5  Unsure………..…99 | Owner operated…………....1  Rented in (cash)……………2  Sharecropped in……………3  Borrowed (no payment)…...4  Rented out (cash)………….5  Lending out (no payment)...6  Other (specify)….………….7  Unsure…… ……………….99 | Agriculture/crop production……….1  Grazing for livestock………………2  Homestead/ house plot…………...3  Bush………………………………...4  Commercial/non ag enterprise…...5  Fallow……………………………….6  Other (specify)……………………..7  Unsure ……………………………99 | Good……………1  Medium…………2  Poor……………..3  Unsure …………99 | Rain fed…………1  Pond-irrigation…2  Tube well ………3  River canal ……4  Diesel pump …..5  Electric pump.....6  Others …………7  Unsure ……….99 | ***See final page*** |

# Module D: Vegetable production, costs and revenues

**VEGETABLE production by crop in 2018-19 crop year** **(IMPORTANT: use one row for each vegetable grown, recording information on that vegetable for the whole year and in aggregate across all plots. So if the same vegetable was grown on multiple plots and/or multiple times in the year, ask for total across plots and for the whole year.**

How many vegetables types did you grow in the last year? _______________

**Enumerator read:** Now let us think in detail about each vegetable that you grew last year. I am going to ask you a series of questions about your vegetable production in detail. Once again, this is for the period starting from Kharif-1 until and including Rabi 2018-19 i. e Kharif-1 (16 March-15 July 2018), Kharif-2 (16 July-15 November, 2018) and Rabi (16 November, 2018-15 March 2019.

**Respondent ID: ______ (from Module B)**

**D.1. Characteristics of the plot and harvest**

| Name of vegetables grown | Cropping area  Hectares…….…1  Square meters…2  Acres…………...3  Feet…………….4  Bigha…….…….5  Kattha…….……6  Decimal ............7  Other ……….8 | | Months of planting  (Months 1 to 12) | | | Months of harvest  (Months 1 to 12) | | | Frequency of harvest  1 = daily  2 = every other day  3. = weekly  4 = other | Total quantity of [VEGETABLE] actually harvested during the year  1=kilograms  2=quintals  3=tonnes | |
| --- | --- | --- | --- | --- | --- | --- | --- | --- | --- | --- | --- |
| **Code 1** | Area | Unit code (above) | Plant 1 | Plant 2 | Plant 3 | Harvest 1 | Harvest 2 | Harvest 3 | Code (above) | Quantity | Unit code (above) |
| D101 | D102a | D102b | D103a | D103b | D103c | 104a | 104b | 104c | D105 | D106a | D106b |
|  |  |  |  |  |  |  |  |  |  |  |  |
|  |  |  |  |  |  |  |  |  |  |  |  |
|  |  |  |  |  |  |  |  |  |  |  |  |
|  |  |  |  |  |  |  |  |  |  |  |  |
|  |  |  |  |  |  |  |  |  |  |  |  |
|  |  |  |  |  |  |  |  |  |  |  |  |
|  |  |  |  |  |  |  |  |  |  |  |  |

**D.2 Disposal of harvested produce: how was the harvested produce in column D106 disposed of?**

| Sold | | | | | | | Eaten at home  Quantity  1=kilograms  2=quintal  3=tonnes | | Given away  Quantity  1=kilograms  2=quintals  3=tonnes | | Wasted  Quantity  1=kilograms  2=quintals  3=tonnes | |
| --- | --- | --- | --- | --- | --- | --- | --- | --- | --- | --- | --- | --- |
| Vegetable code | Total quantity  sold | Unit  1=kilograms  2=quintals  3=tonnes | % sold to LOOP | % sold to other sources | Earnings from LOOP | Earnings from others |  |  |  |  |  |  |
| **CODE 1** | Units | Unit code (above) | **Code** (above) | % | Tk. | Tk. | Units | Unit Code  (above) | Units | Unit Code  (above) | Units | Unit Code (above) |
| D201a | D201b | D201c | D201d | D201e | D201f | D201g | D202a | D202b | D203a | D203b | D204a | D204b |
|  |  |  |  |  |  |  |  |  |  |  |  |  |
|  |  |  |  |  |  |  |  |  |  |  |  |  |
|  |  |  |  |  |  |  |  |  |  |  |  |  |
|  |  |  |  |  |  |  |  |  |  |  |  |  |
|  |  |  |  |  |  |  |  |  |  |  |  |  |
|  |  |  |  |  |  |  |  |  |  |  |  |  |
|  |  |  |  |  |  |  |  |  |  |  |  |  |

**D.3. Input use**

| Veg code | Own or exchanged seeds | Purchased seeds | | Chemical fertilizers  (Urea, TSP, MP, Zinc, S, etc.) | | Pesticide/Insecticide/Fungicide | | Plant Growth hormone | | Cost of irrigation | | | Organic manure – compost – oilcakes-Cow dung | | |
| --- | --- | --- | --- | --- | --- | --- | --- | --- | --- | --- | --- | --- | --- | --- | --- |
|  |  |  |  |  |  |  |  |  |  | Number of hours | | Expenditures | Number of kgs | | Expenditures |
| **CODE 1** | Kgs | Kgs | Tk. | Kgs | Tk. | Litres/kg | Tk. | Litres/Kg | Tk. | Own | Purchased | Tk. | Own | Purchased | Tk. |
| D301 | D301 | D302a | D302b | D303a | D303b | D304a | D304b | D305a | D305b | D306a | D306b | D306c | D307a | D307b | D307c |
|  |  |  |  |  |  |  |  |  |  |  |  |  |  |  |  |
|  |  |  |  |  |  |  |  |  |  |  |  |  |  |  |  |
|  |  |  |  |  |  |  |  |  |  |  |  |  |  |  |  |
|  |  |  |  |  |  |  |  |  |  |  |  |  |  |  |  |
|  |  |  |  |  |  |  |  |  |  |  |  |  |  |  |  |
|  |  |  |  |  |  |  |  |  |  |  |  |  |  |  |  |
|  |  |  |  |  |  |  |  |  |  |  |  |  |  |  |  |
|  |  |  |  |  |  |  |  |  |  |  |  |  |  |  |  |

**D.4. Labour use**

| Vegetable code | Soil preparation-ploughing  (number of man-days*)  *Man-days = people x number of days work | | | | Planting/Rouging (i.e. pick out bad plants)  (number of man-days) | | | | Hilling  (number of man-days) | | | | Weeding  (number of man-days) | | | | |
| --- | --- | --- | --- | --- | --- | --- | --- | --- | --- | --- | --- | --- | --- | --- | --- | --- | --- |
| **CODE 1** | Family | % of family labour that is female | Wage labour | % wage labour that is female | Family | % of family labour that is female | Wage labour | % wage labour that is female | Family | % of family labour that is female | Wage labour | % wage labour that is female | Family | % of family labour that is female | Wage labour | % of wage labour that is female | |
|  | D401a | D401b | D401c | D401d | D402a | D402b | D402c | D402d | D403a | D403b | D403c | D403d | D404a | D404b | D404c | D404d | |
|  |  |  |  |  |  |  |  |  |  |  |  |  |  |  |  |  | |
|  |  |  |  |  |  |  |  |  |  |  |  |  |  |  |  |  | |
|  |  |  |  |  |  |  |  |  |  |  |  |  |  |  |  |  | |
|  |  |  |  |  |  |  |  |  |  |  |  |  |  |  |  |  | |
|  |  |  |  |  |  |  |  |  |  |  |  |  |  |  |  |  | |
|  |  |  |  |  |  |  |  |  |  |  |  |  |  |  |  |  | |
|  |  |  |  |  |  |  |  |  |  |  |  |  |  |  |  |  | |
|  |  | | | |  | | | |  | | | |  | | | | |
| Vegetable code | Watering  (number of man-days) | | | | Fertilizer/Insecticides/Pesticides/Fungicides/Hormone ((number of man-days)) | | | | Harvesting  (Number of man-days) | | | | Other activities  (Number of days) | | | | |
| **CODE 1** | Family  labour | % of family labour that is female | Wage labour | % wage labour that is female | Family  labour | % of family labour that is female | Wage labour | % wage labour that is female | Family  labour | % of family labour that is female | Wage labour | % wage labour that is female | Family | % of family labour that is female | Wage labour | | % wage labour that is female |
|  | D405a | D405b | D405c | D405d | D406a | D406b | D406c | D406d | D407a | D407b | D407c | D407d | D408a | D408b | D408c | | D408d |
|  |  |  |  |  |  |  |  |  |  |  |  |  |  |  |  | |  |
|  |  |  |  |  |  |  |  |  |  |  |  |  |  |  |  | |  |
|  |  |  |  |  |  |  |  |  |  |  |  |  |  |  |  | |  |
|  |  |  |  |  |  |  |  |  |  |  |  |  |  |  |  | |  |
|  |  |  |  |  |  |  |  |  |  |  |  |  |  |  |  | |  |

**D5. Technology use**

| Vegetable code | Months of harvest (1-12) | Animal Plough | | | Power Tiller/Tractor/harvester/thresher use | | | Use of *Rotavator* | | |
| --- | --- | --- | --- | --- | --- | --- | --- | --- | --- | --- |
| **CODE 1** | Month | Number of hours | | Expenses | Number of hours | | Expenses | Number of hours | | Expenses |
|  |  | Own | Rented | Tk. | Own | Rented | Tk. | Own | Rented | Tk. |
| D501 | D502 | D503a | D503b | D503c | D504a | D504b | D504c | D505a | D505b | D505c |
|  |  |  |  |  |  |  |  |  |  |  |
|  |  |  |  |  |  |  |  |  |  |  |
|  |  |  |  |  |  |  |  |  |  |  |
|  |  |  |  |  |  |  |  |  |  |  |
|  |  |  |  |  |  |  |  |  |  |  |
|  |  |  |  |  |  |  |  |  |  |  |

**Codes for Module D: Vegetable production**

| **Code 1 (D101) Crop types** |
| --- |
| **See final page** |

# Module E. Marketing of Vegetables

**Respondent ID: ______ (from Module B)**

**Enumerator read:** We would now like to find out more about your vegetable sales. Here we seek information over the last 12 months – **March 2018 to now**

**E.1. Aggregated transactions**

**E101:**

| a. Number of times (days) in the last 12 months in which you sold any vegetable | b. Of these, how many were to LOOP? | c. Number of times (days) in Rabi season (16 November 2018 to 15 March 2019) in which you sold vegetables | d. Of these, how many were to LOOP? | e. Number of times (days) in the last Kharif-2 (16 July to 15 November 2018) season in which you sold vegetable | f. Of these, how many were to LOOP? | g. Number of times (days) in the last Kharif-1 (16 March to 15 July 2018) season in which you sold vegetable | h. Of these, how many were to LOOP? |
| --- | --- | --- | --- | --- | --- | --- | --- |
|  |  |  |  |  |  |  |  |

Enumerator read: Now we would like to understand the details of a typical vegetable sales transaction. Please think about details of the last occasion when you sold vegetables to LOOP that you consider typical for the Rabi

Did you sell Vegetables through LOOP aggregation in the last Rabi season……………………..Yes……………….No.

**E.2. Last LOOP transaction**

Please complete for the **LAST LOOP TRANSACTION**

| Month of sale  (1-12) | How many people did you sell to on this date?  (e.g. Retailers, Aratdars) | First buyer (the one you sold to) | List up to three vegetable items sold – top 3 veg. included in this transaction | | | | | | Total quantity of veg sold  [all veg in the transaction] | Price received veg 1 | Price received veg 2 | Price received veg 3 | Total amount received | Major reason for the choice of this buyer | Did you receive advanced price information from LOOP before the transaction?    1. yes  2 no |
| --- | --- | --- | --- | --- | --- | --- | --- | --- | --- | --- | --- | --- | --- | --- | --- |
|  |  |  | Veg 1  Code  (top 3) | Veg 2  Code | Veg 3 Code | Quantity of veg 1 sold | Quantity of veg 2 sold | Quantity of veg 3 sold |  |  |  |  |  |  |  |
| Month | Number | **CODE 1** | **CODE 1** MODULE D | **CODE 1** MODULE D | **CODE 1** MODULE D | Quintals | Quintals | Quintals | Quintals | Tk./kg | Tk./kg | Tk/kg | Tk. | **CODE 2** | Code (above) |
| E201 | E202 | E203 | E204a | E204b | E204c | E205a | E205b | E205c | E206 | E207a | E207b | E207c | E207d | E208 | E209 |
|  |  |  |  |  |  |  |  |  |  |  |  |  |  |  |  |

| Sale location (where did the aggregator travel to?) | Market distance | Total monetary deductions from LOOP sale | | Deduction type 1 | Deduction type 2 | Deduction type 3 | Deduction incurred type 1 amount | Deduction incurred type 2 amount | Deduction incurred type 3 amount | Modes of payment | % paid cash and immediately | If credit, length of credit (days after delivery to buyer) | Receipt obtained? |
| --- | --- | --- | --- | --- | --- | --- | --- | --- | --- | --- | --- | --- | --- |
| **CODE 3** | km | Amount | Unit  **CODE 5** | **CODE 6** | **CODE 6** | **CODE 6** | Unit as per E212b | Unit as per E212b | Unit as per E212b | **CODE 7** | % | Number of days | 1. Yes  2. No |
| E210 | E211 | E212a | E212b | E213a | E213b | E213c | E214a | E214b | E214c | E215 | E216 | E217 | E218 |
|  |  |  |  |  |  |  |  |  |  |  |  |  |  |

E219: Did this last transaction take place during the peak marketing time of the last Rabi season (e.g. during the week with the highest volumes of vegetable sales)? ________ (Y = 1 🡪 proceed to Section E.4; N = 0 🡪 E220)

E220: If not, when was your peak marketing time during the last Rabi season? __________ (week: 1-4); (b) __________ (month: 1-12)

E221: Approximately how much more would the average transaction be during the peak period relative to your last transaction (i.e. detailed from E201 to E218) _________ (difference); (b) __________ (unit: 1 – kg, 2 – quintal, 3 - %)

**E.3. Last non-LOOP transaction**

| Month of sale  (1-12) | How many people did you sell to on this date?  (e.g. Retailers, Aratdars) | First buyer (the one you sold to) | List up to three vegetable items sold – top 3 veg. included in this transaction | | | | | | Total quantity of veg sold  [all veg in the transaction] | Price received veg 1 | Price received veg 2 | Price received veg 3 | Total amount received | Major reason for the choice of this buyer | Did you contact the potential buyer in advance?    1. yes  2 no |
| --- | --- | --- | --- | --- | --- | --- | --- | --- | --- | --- | --- | --- | --- | --- | --- |
|  |  |  | Veg 1  Code  (top 3) | Veg 2  Code | Veg 3 Code | Quantity of veg 1 sold | Quantity of veg 2 sold | Quantity of veg 3 sold |  |  |  |  |  |  |  |
| Month | Number | CODE 1 | CODE 1 MODULE D | CODE 1 MODULE D | CODE 1 MODULE D | Quintals | Quintals | Quintals | Quintals | Tk/kg | Tk./kg | Kg./kg | Tk | CODE 2 | Code (above) |
| E301 | E302 | E303 | E304a | E304b | E304c | E305a | E305b | E305c | E306 | E307a | E307b | E307c | E307d | E308 | E309 |
|  |  |  |  |  |  |  |  |  |  |  |  |  |  |  |  |

| Sale location | Market distance | Trans-action time at the location of sale | Time between departure home and arrival location sale | Transport means | Transport costs | Quantity wasted during the marketing | Other transaction cost incurred type 1 | Other transaction cost incurred type 2 | Other transaction cost incurred type 1  amount | Other transaction cost incurred type 2 amount | Input advance? | Payment mode | % paid cash and immediately | If credit, length of credit (days after delivery to buyer) | Receipt obtained? |
| --- | --- | --- | --- | --- | --- | --- | --- | --- | --- | --- | --- | --- | --- | --- | --- |
| CODE 3 | km | 24hr clock | Round up to a nearest hour | CODE 4 | Tk. | Kg | CODE 6 | CODE 6 | % | % | 1. Yes  2. No | CODE 7 | % | Number of days | 1. Yes  2. No |
| E310 | E311 | E312a | E312b | E313 | E314 | E315 | E316a | E316b | E317a | E317b | E318 | E319 | E320 | E321 | E322 |
|  |  |  |  |  |  |  |  |  |  |  |  |  |  |  |  |

E323: Did this last transaction take place during the peak marketing time of the last Rabi season (e.g. during the week with the highest volumes of vegetable sales)? _____(Y = 1 🡪 proceed to Section E.4; N = 0 🡪 E324)

E324: If not, when was your peak marketing time during the last Rabi season? __________ (week: 1-4); (b) __________ (month: 1-12)

E325: Approximately how much more would the average transaction be during the peak period relative to your last transaction (i.e. detailed from E301 to E322) ______ (difference); (b) ______ (unit: 1 – kg, 2 – quintal, 3 - %)

**E.4. Last LOOP transaction during the Kharif-2 season (**16 July-15 November **2018)**

E401a: Was the LOOP transaction in E.1 during the Kharif-2 season? _________ (Yes = 1 🡪 proceed to section F; No = E401b)

E401b: If no to above: did you sell any vegetables to LOOP in the Kharif 2 season? _________ (Yes = 1 🡪 E402; No = 0 🡪 next section)

Please complete for the **LAST LOOP TRANSACTION in Kharif-2**

| Month of sale  (1-12) | How many people did you sell to on this date?  (e.g. Retailers, Aratdars) | First buyer (the one you sold to) | List up to three vegetable items sold – top 3 veg. included in this transaction | | | | | | Total quantity of veg sold  [all veg in the transaction] | Price received veg 1 | Price received veg 2 | Price received veg 3 | Total amount received | Major reason for the choice of this buyer | Did you receive advanced price information from LOOP before the transaction?    1. yes  2 no |
| --- | --- | --- | --- | --- | --- | --- | --- | --- | --- | --- | --- | --- | --- | --- | --- |
|  |  |  | Veg 1  Code  (top 3) | Veg 2  Code | Veg 3 Code | Quantity of veg 1 sold | Quantity of veg 2 sold | Quantity of veg 3 sold |  |  |  |  |  |  |  |
| Month | Number | CODE 1 | CODE 1 MODULE D | CODE 1 MODULE D | CODE 1 MODULE D | Quintals | Quintals | Quintals | Quintals | Tk./kg | Tk./kg | Tk./kg | Tk. | CODE 2 | Code (above) |
| E402 | E403 | E404 | E405a | E405b | E405c | E406a | E406b | E406c | E406d | E407a | E407b | E407c | E407d | E408 | E409 |
|  |  |  |  |  |  |  |  |  |  |  |  |  |  |  |  |

| Sale location (where did the aggregator travel to?) | Market distance | Total monetary deductions from LOOP sale | | Deduction type 1 | Deduction type 2 | Deduction type 3 | Deduction incurred type 1 amount | Deduction incurred type 2 amount | Deduction incurred type 3 amount | Modes of payment | % paid cash and immediately | If credit, length of credit (days after delivery to buyer) | Receipt obtained? |
| --- | --- | --- | --- | --- | --- | --- | --- | --- | --- | --- | --- | --- | --- |
| CODE 3 | km | Amount | Unit  CODE 5 | CODE 6 | CODE 6 | CODE 6 | Unit as per E412b | Unit as per E412b | Unit as per E412b | CODE 7 | % | Number of days | 1. Yes  2. No |
| E410 | E411a | E412a | E412b | E413a | E413b | E413c | E414a | E414b | E414c | E415 | E416 | E417 | E418 |
|  |  |  |  |  |  |  |  |  |  |  |  |  |  |

E419: Did this last transaction take place during the peak marketing time of the last Kharif-2 season (e.g. during the week with the highest volumes of F&V sales)? ________ (Y = 1 🡪 proceed to next module; N = 0 🡪 E420)

E420: If not, when was your peak marketing time during the last Kharif-2 season? __________ (week: 1-4); (b) __________ (month: 1-12)

E421: Approximately how much more would the average transaction be during the peak period relative to your last transaction (i.e. detailed from E401 to E418) _________ (difference); (b) __________ (unit: 1 – kg, 2 – quintal, 3 - %)

**E.5. Last LOOP transaction during the Kharif-1 season (16 March-15 July, 2018)**

E501a: Was the LOOP transaction in E.1 during the Kharif-1 season? _________ (Yes = 1 🡪 proceed to section F; No = E501b)

E501b: If no to above: did you sell any vegetables to LOOP in the Kharif-1 season? _________ (Yes = 1 🡪 E502; No = 0 🡪 next section)

Please complete for the **LAST LOOP TRANSACTION in Kharif-1**

| Month of sale  (1-12) | How many people did you sell to on this date?  (e.g. Retailers, Aratdars) | First buyer (the one you sold to) | List up to three F&V items sold – top 3 vegs. included in this transaction | | | | | | Total quantity of veg sold  [all veg in the transaction] | Price received veg 1 | Price received veg 2 | Price received veg 3 | Total amount received | Major reason for the choice of this buyer | Did you receive advanced price information from LOOP before the transaction?    1. yes  2 no |
| --- | --- | --- | --- | --- | --- | --- | --- | --- | --- | --- | --- | --- | --- | --- | --- |
|  |  |  | Veg 1  Code  (top 3) | Veg 2  Code | Veg 3 Code | Quantity of veg 1 sold | Quantity of veg 2 sold | Quantity of veg 3 sold |  |  |  |  |  |  |  |
| Month | Number | CODE 1 | CODE 1 MODULE D | CODE 1 MODULE D | CODE 1 MODULE D | Quintals | Quintals | Quintals | Quintals | Tk./kg | Tk./kg | Tk./kg | Tk. | CODE 2 | Code (above) |
| E502 | E503 | E504 | E505a | E505b | E505c | E506a | E506b | E506c | E506d | E507a | E507b | E507c | E507d | E508 | E509 |
|  |  |  |  |  |  |  |  |  |  |  |  |  |  |  |  |

| Sale location (where did the aggregator travel to?) | Market distance | Total monetary deductions from LOOP sale | | Deduction type 1 | Deduction type 2 | Deduction type 3 | Deduction incurred type 1 amount | Deduction incurred type 2 amount | Deduction incurred type 3 amount | Modes of payment | % paid cash and immediately | If credit, length of credit (days after delivery to buyer) | Receipt obtained? |
| --- | --- | --- | --- | --- | --- | --- | --- | --- | --- | --- | --- | --- | --- |
| CODE 3 | km | Amount | Unit  CODE 5 | CODE 6 | CODE 6 | CODE 6 | Unit as per E412b | Unit as per E412b | Unit as per E412b | CODE 7 | % | Number of days | 1. Yes  2. No |
| E510 | E511a | E512a | E512b | E513a | E513b | E513c | E514a | E514b | E514c | E515 | E516 | E517 | E518 |
|  |  |  |  |  |  |  |  |  |  |  |  |  |  |

E519: Did this last transaction take place during the peak marketing time of the last Kharif-1 season (e.g. during the week with the highest volumes of F&V sales)? ________ (Y = 1 🡪 proceed to next module; N = 0 🡪 E520)

E520: If not, when was your peak marketing time during the last Kharif-1 season? __________ (week: 1-4); (b) __________ (month: 1-12)

E521: Approximately how much more would the average transaction be during the peak period relative to your last transaction (i.e. detailed from E501 to E518) _________ (difference); (b) __________ (unit: 1 – kg, 2 – quintal, 3 - %)

**Codes for Module E: Marketing of vegetables**

| **CODE 1: First buyer** | | **CODE 2: Reason for buyer** | **CODE 3: Sale location** | | **CODE 4: Transport means** | **CODE 5: Deduction units** | **CODE 6: Deduction/ transaction cost type** | **CODE 7: Payment modes** |
| --- | --- | --- | --- | --- | --- | --- | --- | --- |
| Another local wholesaler in village…..1  Another local wholesaler from outside…2  Wholesaler in market…3  Retailers in market 4  NGO……………..5 | Exporter..6  Cooperative society…7  Traditional retailer……..8  Supermarket.....9  Consumer……10  Hotel/restaurant..11 | Good prices……1  Accept large quantities……2  Accept small quantities……3  Gives advances.4  Pays quickly…...5  Close by………..6  Good communication 7  Low market charges…….8.  No other choice.9 | Farmer’s field or own village……1  Local Wholesale market ……2  Wholesale market. in this district…….3  Other distance wholesale market …….4 | Local retail market…5  Cold storage……...6  Other (specify)….7 | Porter/own …….1  Rickshaw/Van………2  Tricycle Van……3  Hand-cart………4  Alamshadhu……5  Nachiman………6.  Kariman…………7.  Pick-up………….8  Truck……………9  Bicycle………….10  Motorbike...........11  Other (specify).12 | Tk, …………1  Tk/kg ……………..2  Tk/quintal ……….3  Tk/km ……………5  Paisa/km …………6  Paisa ……………..7  Paisa/kg…………..8  Paisa/quintal .……9  % sale…………….10  Other …………….11 | LOOP transport costs …1  Bagging/boxing…………2  Loading/off………………3  Payments at checkpoint or road-block ………..4  Personal transport to/from wholesale market …..5  Entry license fee at the market ……………….6  Aratdar commission….7  Weighing fees…….........8  Grading………………….9  Bribe/Toll……………….......10  Other……………………11 | In cash……………….1  In-kind (e.g. agri input)……………...2  Partly in cash, partly in kind………………..3  Cheque………………4  Mobile cash………….5.  Others………………..6 |

# Module F: Revenues and costs from all other crops, and livestock holdings

**Respondent ID _______ (Module B)**

**Total crop production, revenues and costs in the last 12 months (excluding vegetables)**

Here we are seeking information on all other crops (other than vegetables grown during the year (Kharif-1, Kharif-2 and Rabi 2018-19). Only one row per crop, and so if the same crop is grown on multiple plots and/or more than once a year, ask for the total across plots and for the whole year

**F.1. Crop production and use**

| Crop  CODE 1 | Area cultivated | Main month of planting | Main month of harvest | Quantity harvested  1=kilograms  2=quintals  3=tonnes | | Quantity sold  1=kilograms  2=quintals  3=tonnes | | Average price received |
| --- | --- | --- | --- | --- | --- | --- | --- | --- |
|  | **CODE 2** | (1-12) | (1-12) | Quantity | **Code above** | Quantity | Code above | Tk./kg |
|  | F101 | F102 | F103 | F104a | F104b | F105a | F105b | F106 |
| 1. |  |  |  |  |  |  |  |  |
| 2. |  |  |  |  |  |  |  |  |
| 3. |  |  |  |  |  |  |  |  |
| 4. |  |  |  |  |  |  |  |  |
| 5. |  |  |  |  |  |  |  |  |
| 6. |  |  |  |  |  |  |  |  |
| 7. |  |  |  |  |  |  |  |  |

**F.2. Monetary and in-kind or in-barter expenses over the last 12 months (if in kind or in-barter, estimate value)**

| Crop  **CODE 1** | Area cultivated | Ploughing and land preparation costs | Seed costs | Manure costs | Fertilizer | Pesticide/herbicide | Plant growth hormone | Irrigation | Hired labour (Tk) |
| --- | --- | --- | --- | --- | --- | --- | --- | --- | --- |
|  | **CODE 2** | (Tk) | (Tk) | (Tk) | (Tk) | (Tk) | (Tk) | (Tk) | (Tk) |
|  | F201 | F202 | F203 | F204 | F205 | F206 | F207 | F208 | F209 |
| 1. |  |  |  |  |  |  |  |  |  |
| 2. |  |  |  |  |  |  |  |  |  |
| 3. |  |  |  |  |  |  |  |  |  |
| 4. |  |  |  |  |  |  |  |  |  |
| 5. |  |  |  |  |  |  |  |  |  |
| 6. |  |  |  |  |  |  |  |  |  |
| 7. |  |  |  |  |  |  |  |  |  |

**F.3 Livestock and Fisheries**

**Respondent ID _______ (Module B)**

| ID | LIVESTOCK and FISHERIES | How many [LIVESTOCK/FISHERIES] does the household own today?  **Enter number**  If 0 >>  next type | What is the main purpose of owning [LIVESTOCK/FISHERIES]?  Consumption = 1  Income = 2  Farming = 3 |
| --- | --- | --- | --- |
| **ID** |  | **F401** | **F402** |
| A | Cattle |  |  |
| B | Buffalo |  |  |
| C | Goats & sheep |  |  |
| D | Chickens |  |  |
| E | Ducks |  |  |
| F | Pigeons |  |  |
| G | Other (specify) |  |  |

**Code for Module F: Marketing of other crops, livestock, and fisheries**

| **Code 1: Crop, livestock and fisheries types** | **Code 2: area cultivated** |
| --- | --- |
| See final page | Hectares…………….1  Square meters……...2  Acres…………………3  Feet…………………..4  Bigha…………………5  Kattha………………..6  Decimal………………7  Unsure ……………...99 |

# module G: Non-CROP income in the last 12 months

**Enumerator read:** Now we are going to ask you about your other sources of income. In each case, please think of total earning/revenue over the last 12 months, as well as the costs associated with the livelihood activity. Then provide an estimate of the NET INCOME (revenue/earning minus costs) over the last 12 months.

**Respondent ID: ________ (Module B)**

**G.1. Other income in the last 12 months**

|  | Source | Unit | In the last 12 months |  | Source | Unit | In the last 12 months |
| --- | --- | --- | --- | --- | --- | --- | --- |
| G101 | Wage labour/salary income | Tk. |  | G108 | Income from selling a house | Tk. |  |
| G102 | Net income from fishery | Tk. |  | G109 | Income from selling land | Tk. |  |
| G103 | Net income from dairy | Tk. |  | G110 | Income from selling durable consumable goods | Tk. |  |
| G104 | Rental income of leased-out land | Tk. |  | G111 | Net income from other family business or enterprise | Tk. |  |
| G105 | Other rental income | Tk. |  | G112 | Net income from poultry | Tk. |  |
| G106 | Interest of deposits/pension | Tk. |  | G113 | Other 1 | Tk. |  |
| G107 | Interest from lending | Tk. |  | G114 | Other 2 | Tk. |  |

**G.2. Remittances**

In the last 12 months, have you or any member of the household received any money from any person who does not live in your household? __________ *1. Yes, 2. No (if No, go to H)*

| What is the relationship of the [Benefactor] to the household head?  *(1=Son/daughter/father/mother; 2=Other relatives; 3=Non-relatives)* | | | How much money in total did your household receive from the [Benefactor] in the last 12 months? | | |
| --- | --- | --- | --- | --- | --- |
| (Write relationship code) | | | (Tk) | | |
| G201 | G202 | G203 | G204 | G205 | G206 |
| Donor 1 | Donor 2 | Donor 3 | Donor 1 | Donor 2 | Donor 3 |
|  |  |  |  |  |  |

# Module H: Assets

**Enumerator read:** We would now like to ask you about your household and agricultural assets to build a picture of the agricultural technology at your disposal. Please think about the number of the following assets you own today and this time approximately two years ago (e.g. 2017)

**Respondent ID: ________ (Module B)**

|  | Equipment | Number owned in household | Two years ago (pre-LOOP) |
| --- | --- | --- | --- |
| **ID** |  | **H101** | **H102** |
| A | Tractor |  |  |
| B | Plough |  |  |
| C | Electric motor (oil engine) |  |  |
| D | Bullock Cart/Trailer |  |  |
| E | Pesticide Sprayer |  |  |
| F | Hoe |  |  |
| G | Seed drill |  |  |
| H | Thresher |  |  |
| I | Shovel/Spade |  |  |
| J | Winnower |  |  |
| K | Combine harvester |  |  |
| L | Power Tiller |  |  |
| M | Weeder |  |  |
| N | Storage shed/Godown |  |  |
| O | Livestock shed |  |  |
| P | Bicycle |  |  |
| Q | Motorbike |  |  |
| R | Car |  |  |
| S | Television |  |  |
| T | CD/Cassette player/radio |  |  |
| U | Fridge |  |  |
| V | Mobile phone |  |  |
| W | Cooking stove |  |  |
| X | Sewing machine |  |  |
| Y | Computer/laptop |  |  |

# Module I: Housing (for wealth index)

I101. What is the construction material of the wall and of the roof of the house in which you live? A. Wall: ____, _____; B. Roof: _____, _____.

*1. Bricks – burnt; 2. Stones; 3. RCC; 4. Concrete / Cement Concrete; 5. GI tin /metal sheets; 6. Asbestos Cement; 7. Bamboo; 8. Leaf / Branch / Grass Reeds; 9. Wood / Timber; 10. Thatch / Mud; 11. Plastic / Canvas; 12. Others: _______*

I102. What is the main material of the floor? _________ *1.cement, 2.mud, 3.wood, 4.sand, 5.other (specify________________)*

I103. How many rooms does your household occupy? _________

I104. Do you own the dwelling you reside in? _______*1.Yes 2.No [If Yes, Go to Question 7]*

I105. Do you pay any rent for this dwelling? _______*1.Yes 2.No*

I106. If yes, how much do you pay per month? ______ Tk. per month *[Include cash payment plus the value of in-kind payments, if any]*

I107. If you own the house, what is the estimated value of your house? _______________ Tk.

I108. What is your main source of light now? _____ Two years ago? _______ *1. Kerosene; 2. Candle; 3. Electricity; 3. Electricity with inverter; 5. Solar energy; 6. LPG/biogas; 7. Battery (torch); 8. Diesel generator; 9. Other, specify_____*

I109. What is your main source of heat for cooking now? _____ Two years ago? _________*1. Wood/charcoal; 2. Kerosene; 3. Electricity; 4. LPG/biogas; 5. Crop residues; 6. Other, specify_____*

I110. What is your main source of drinking water? _____ Two years ago? __________*1. River/canal; 2. Public well; 3. Public handpump; 4. Own handpump; 5. Own motor pump or other water-lifting devices; 6. Piped water; 7. Rainwater; 8. Water filling station; 9. Other, specify: ______*

# Module J: Pesticide safety: knowledge, attitudes, practices

**Respondent ID: ________ (Module B)**

J101: Which crop category is sprayed most? (1=vegetables; 2=staple cereals (wheat, rice); 3=pulses/millet/sorghum; 4=cash crops (cotton, Jute *etc.*); 5=other)

J102: Which vegetable category is sprayed most? (1=green leafy vegetables (*eg. spinach, cabbage, lettuce)*; 2-cauliflower; 3=brinjal; 4=tomato; 5=chilli; 6=gourds; 7=ladies finger; 8=other; 9= all sprayed equally)

J103: Which of the following most closely characterises your approach to spraying?

1= we spray routinely, according to a set schedule in the crop calendar

2= we spray in a reactive mode, based on observations of emerging pest problems.

J104: Typically, how many times in a growing season do you spray vegetable crops? -------------------

J.105: Why do you spray? 1= to control insect; 2=to control disease; 3=others (i.e., growth hormone)

J106: Who is the main person responsible for spraying/applying? -------------- (1=head of household; 3=spouse of household head; 4=son; 5=daughter; 6=other relative; 7=hired worker; 8=other)

J107: Does the sprayer read the contents of the pesticide bottle before applying? 1=always; 2=sometimes; 3=never;

J108: Exposure to the pesticide can have an adverse impact on human health (1=agree; 2=disagree; 3=not sure)

J109: How many days you wait to harvest vegetable after spraying? (For sale and own consumption): For (1) sale; .....................days; and (2) own consumption...............days

J110: Which of the following are intake pathways for pesticides: (1=yes; 2=no; 3=don’t know)

a) Breathing in pesticide ___________

b) Being bitten by a mosquito _________

c) Getting pesticide on skin _________

d) Swallowing pesticide ________
e) Consuming foods from farms that spray pesticide heavily _______

J111: Pesticide containers can be reused safely after cleaning: (1=agree; 2=disagree; 3=not sure)

J112: Does the pesticide sprayer routinely wear any of the following protective clothing while spraying (1=Yes; 2=No; 3=not sure):

i) Long sleeved shirt ­­­­______________

ii) Gloves ­­­­­­­­­­­­­­__________________

iii) Hat or other head cover _______________

iv) Scarf/handkerchief to cover face ___________

v) Rubber boots ________________

J113: Have you received training about the safe use of pesticide? 1=yes; 2=no; 3=not sure.

# Module K: Networks and infrastructure

**Respondent ID: _________ (see module B)**

K101: Is your household a member of Digital Green’s self-help group? ____ *1. Yes; 2. No*

K102: Is your household a member of Micro-credit or any other financial group? ____ *1. Yes; 2. No*

K103: If yes, for how many Micro-credit and other financial groups? ______

K104: Is your household a member of any input and extension service providers (Govt/Non-Govt)? ____ *1. Yes; 2. No*

K105: Is your household a member of any other farmers’ organizations/producer’s group ___ 1*. Yes; 2.No*

K106: Is your household a member of any other cooperative ____1. Yes, 2. No

K107: How many other farm households in your village do you know that have supplied vegetables to LOOP in the past? ________ (Number)

K108: Please think about your five nearest neighbours (by distance from home). How many of them have supplied to LOOP in the past? _______ (Number) or 9=don't know.

**K.2. Access to infrastructure**

|  |  | How far (in km) is the household located from the nearest of the following infrastructure: |
| --- | --- | --- |
| K201 | Paka/Pave road |  |
| K202 | LOOP aggregation point |  |
| K203 | Agricultural inputs market/shop |  |
| K204 | Village *haat/Bazar* |  |
| K205 | Major agricultural market |  |
| K206 | School |  |
| K207 | Hospital |  |
| K208 | Farmers’ cooperative office |  |
| K209 | Union office |  |
| K210 | Upazila agriculture office |  |

**K.3. Perceived quality of road infrastructure**

|  |  | What are the condition of the roads to the following locations?  (very good = 1, good =-2, average = 3, poor = 4, very poor = 5) |
| --- | --- | --- |
| K301 | LOOP aggregation point |  |
| K302 | Agricultural inputs market/shop |  |
| K303 | Village *haat/Bazar* |  |
| K304 | Major agricultural market |  |
| K305 | Upazila agriculture office |  |

# Module L: Loop participation and adoption (for Loop farmers)

**Respondent ID: ________ (Module B)**

**Peer effects**

L101: When did you first supply LOOP? ________ (mm/yyyy)

L102: Where did you first learn about LOOP? ________ (CODE 1)

L103a: Were you actively encouraged to join LOOP? ________ (N = 0 🡪 L104, Y = 1 🡪 L103b)

L103b: If yes, who actively encouraged you to join LOOP? ________ (CODE 1)

L104: How many of your farming peers have supplied LOOP in the past year? ________ (number) (0 🡪 L106, >0 🡪 L105)

L105: If >0, what was the earliest date that one of your peers supplied LOOP? ____________ (mm/yyyy)

L106: How often do you discuss farming practices and/or marketing activities with your farming peers? __________ (CODE 2)

L107: Have you actively encouraged others to join LOOP? ________ (N = 0, Y = 1)

**Effectiveness of extension efforts**

L108: Prior to joining, how many non-farmers told you about LOOP? __________ (number)

L109: Did you attend any LOOP village extension meetings prior to joining LOOP? __________ (N = 0, Y = 1)

L110: Were there any familiarisation or training sessions prior to you joining LOOP (N = 0, Y = 1)

L111: Which of the following factors had the greatest influence on you joining LOOP? __________ (CODE 3)

| **Perceived benefits of LOOP participation** | | | | | **Perceived challenges of LOOP participation** | | | | | **Continuation of LOOP participation** | |
| --- | --- | --- | --- | --- | --- | --- | --- | --- | --- | --- | --- |
| Rate the extent to which you agree with these benefits of LOOP participation (1 – strongly disagree; 5 – strongly agree). Then rank the top two benefits. | | | | | Rate the extent to which you agree with the following challenges of LOOP participation (1 – strongly disagree; 5 – strongly agree i.e. LOOP is a challenge) | | | | | Will you continue to supply LOOP over the next year?  Yes=1 >> F31b  No=2 >> G01 | How would you anticipate the frequency of LOOP participation to change over the next year?  1=increase  2=decrease  3=stay the same |
| LOOP has led to obtaining better prices | LOOP has reduced vegetable transport costs | LOOP has led to time-savings | LOOP has led to increased yields | LOOP has led to improved consumption of vegetables at home | Trusting the LOOP aggregator to achieve the best price for your vegetables is a challenge | The cost of LOOP aggregation does not represent value for money | It is a challenge to reach the aggregation location by the designated time | Mobile phone/ LOOP apps use is challenging | The capacity of the LOOP aggregation vehicle is a challenge |  |  |
| L112a | L112b | L112c | L112d | L112e | L113a | L113b | L113c | L113d | L113e | L114a | L114b |
|  |  |  |  |  |  |  |  |  |  |  |  |

**Codes for Module L: LOOP participation and adoption**

| **Code 1 Where hear about LOOP** | **Code** **2 Frequency of farming discussions** | **Code 3 Influence on LOOP adoption** |
| --- | --- | --- |
| Family/friend……………….1  LOOP farmer ………………2  Non-LOOP farmer ………..3  LOOP village meeting…….4  At the market………………5  At seed store……………….6  Observed LOOP aggregation process…………………..7  Self-help-group/Farmers’ cooperative……..............8  Other (specify)……………..9  Unsure …………………..99 | Every day………………………………….1  Multiple times per week………………….2  Once per week……………………………3  Multiple times per month………………...4  Once per month…………………………..5  Less frequently than once per month.....6  Other (specify)……………………………7  Unsure……………………………………99 | Positive experiences of peers ………………..1  LOOP extension activities …………………….2  LOOP training sessions ……………………….3  LOOP familiarisation sessions ………………..4  Costs of alternative approaches (e.g. self-aggregation)…….5  Other (specify) ………………………………….6  Unsure ………………………………………….99 |

# Module M: Consumption and dietary diversity

**Enumerator**: Questions in this and the next section have to be answered by an adult female in the household. This should preferably be the female primary respondent, *eg.* Spouse of the head (if household head is male), or female household head (if household head is female).

**Respondent ID: _________ (Module B)**

**Short food security indicators**

M101: In the past four weeks, how many times did you or any household member have to eat a limited variety of foods due to a lack of resources? ____________ (CODE 1)

M102: In the past four weeks, how many times was there no food to eat of any kind in your household because of lack of resources to get food? ____________ (CODE 1)

M103 In the past four weeks, how many times did you or any household member go a whole day and night without eating anything because there was not enough food? ____________ (CODE 1)

**Dietary diversity**

M104: Have the last 24 hours been part of a special day, like a celebration or feast day or a fast day where you ate special foods or more or less than usual or did not eat because of fasting? _________ (No = 0; Yes, celebration feast day = 1; Yes, celebration fast day = 2). Now please take some time first to carefully think about everything that you ate during the last 24 hours, including breakfast, lunch, dinner, any snacks or tiffin, including meals eaten at home or away.

| Item | **Please record whether the adult female ate any of the following food groups over the past 24 hours**  Food Item | Answer  No = 0  Yes = 1  Unsure = 99 |
| --- | --- | --- |
|  |  |  |
| **ID** |  | **M105** |
| A | Rice (any type including puffed or beaten), roti, peetha, pastry (e.g. grain used in singara), powdered grain mixture (e.g Sattu), other food made from grains |  |
| B | Dal, beans, dried/mature peas (including besan/gram flour), or soybean |  |
| C | White potatoes, white yams, elephant foot (Ol), Haluwa, taro, or any other foods made from starchy roots or tubers |  |
| D | Yellow pumpkin, carrot, sweet potato (orange/yellow inside) |  |
| E | Dark green leafy vegetable |  |
| F | Ripe mango, ripe papaya, ripe jackfruit, ripe litchi |  |
| G | Unripe mango, unripe papaya, unripe jackfruit |  |
| H | Any other fruits, including dried fruits |  |
| I | Any other vegetables |  |
| J | Liver, kidney, heart, or other organ meats |  |
| K | Any meat, such as chicken, duck or other birds, lamb, goat, cow, buffalo |  |
| L | Egg (chicken or duck) |  |
| M | Fresh or dried fish, shellfish, or seafood |  |
| N | Nuts or seeds e.g. peanut, cashew, sunflower, Tisi, Linseed, Sesame, mustard |  |
| O | Paneer or other cheese, yogurt/curd, any kind of animal milk, or other milk products but not including butter, cream, or ice cream |  |
| P | Any food with oil, ghee, fat or butter, including deep-fried or pan-fried foods cooked with oil |  |
| Q | Sugary foods (chocolates, sweets, candies, cake, biscuits), e.g. jilli / jilebi, laddu |  |
| R | Condiments for flavour (chilies, spices, herbs, fish powder, or seeds) |  |
| S | Other beverages and foods (tea or coffee if not sweetened, clear broth) |  |

**Codes for module J consumption and consumption habits**

| **Code 1: (M101-103)** |
| --- |
| 1 = Rarely (1-2 times)  2 = Sometimes (3-10 times)  3 = Often (> 10 times)  99 = Refused |

# Module N: Role in household decision-making

**Enumerator**: This should be answered by the same female who answered the previous section on food consumption.

|  | | | | | | |
| --- | --- | --- | --- | --- | --- | --- |
| “Now I’d like to ask you some questions about your participation in certain types of work activities and on making decisions on various aspects of household life” | | Did you yourself participate in [ACTIVITY] in the past 12 months (that is, during the last [one/two] cropping seasons), from [PRESENT MONTH] last year to [PRESENT MONTH] this year? | When decisions are made regarding [ACTIVITY], who is it that normally takes the decision?  **CIRCLE ALL APPLICABLE**  **IF THE RESPONSE IS SELF ONLY SKIP TO QUESTION G2.05** | How much input did you have in making decisions about [ACTIVITY]?  **USE DECISION CODES FOR G2.03/G2.05;**  **IF NO DECISION MADE, ENTER 98 AND MOVE TO THE NEXT ACTIVITY** | To what extent do you feel you can make your own personal decisions regarding [ACTIVITY] if you want(ed) to?  **CIRCLE ONE** | How much input did you have in decisions on the use of income generated from [ACTIVITY]  **USE CODES FOR G2.03/G2.05** |
| Activity code | Activity description | N101 | N102 | N103 | N104 | N105 |
| A | Food crop farming: These are crops that are grown primarily for household food consumption | YES ............ 1  NO .............. 2  ACTIVITY B | SELF………………………………...1  SPOUSE………………………....…2  OTHER HH MEMBER…………….3  OTHER NON-HH MEMBER……...4  NOT APPLICABLE……………….98   🡪 *NEXT ACTIVITY* |  | NOT AT ALL ............... 1  SMALL EXTENT ......... 2  MEDIUM EXTENT ...... 3  TO A HIGH EXTENT.. 4 |  |
| B | Cash crop farming: These are crops that are grown primarily for sale in the market | YES ............ 1  NO .............. 2  ACTIVITY C | SELF………………………………...1  SPOUSE………………………....…2  OTHER HH MEMBER…………….3  OTHER NON-HH MEMBER……...4  NOT APPLICABLE……………….98   🡪 *NEXT ACTIVITY* |  | NOT AT ALL ............... 1  SMALL EXTENT ......... 2  MEDIUM EXTENT ...... 3  TO A HIGH EXTENT.. 4 |  |
| C | Decisions regarding what to sell when selling food items | YES ............ 1  NO .............. 2  ACTIVITY D | SELF………………………………...1  SPOUSE………………………....…2  OTHER HH MEMBER…………….3  OTHER NON-HH MEMBER……...4  NOT APPLICABLE……………….98   🡪 *NEXT ACTIVITY* |  | NOT AT ALL ............... 1  SMALL EXTENT ......... 2  MEDIUM EXTENT ...... 3  TO A HIGH EXTENT .. 4 |  |
| D | Decisions regarding what to keep for home consumption when selling food items | YES ............ 1  NO .............. 2  ACTIVITY E | SELF………………………………...1  SPOUSE………………………....…2  OTHER HH MEMBER…………….3  OTHER NON-HH MEMBER……...4  NOT APPLICABLE……………….98   🡪 *NEXT ACTIVITY* |  | NOT AT ALL ............... 1  SMALL EXTENT ......... 2  MEDIUM EXTENT ...... 3  TO A HIGH EXTENT .. 4 |  |
| E | Livestock raising | YES ............ 1  NO .............. 2  ACTIVITY F | SELF………………………………...1  SPOUSE………………………....…2  OTHER HH MEMBER…………….3  OTHER NON-HH MEMBER……...4  NOT APPLICABLE……………….98   🡪 *NEXT ACTIVITY* |  | NOT AT ALL ............... 1  SMALL EXTENT ......... 2  MEDIUM EXTENT ...... 3  TO A HIGH EXTENT .. 4 |  |
| **N101/N103 DECISION CODES:**  NO INPUT OR INPUT IN FEW DECISIONS - 01; INPUT INTO SOME DECISIONS- 02; INPUT INTO MOST OR ALL DECISIONS- 03; NO DECISIONS MADE - 98 | | | | | | |

|  |  | Did you yourself participate in [ACTIVITY] in the past 12 months (that is, during the last [one/two] cropping seasons), from [PRESENT MONTH] last year to [PRESENT MONTH] this year? | When decisions are made regarding [ACTIVITY], who is it that normally takes the decision?  **CIRCLE ALL APPLICABLE;**  **IF THE RESPONSE IS SELF ONLY SKIP TO QUESTION G2.05** | How much input did you have in making decisions about [ACTIVITY]?  **USE DECISION CODES FOR G2.03/G2.05**  **IF NO DECISION MADE, ENTER 98** | To what extent do you feel you can make your own personal decisions regarding [ACTIVITY] if you want(ed) to?  **CIRCLE ONE** | How much input did you have in decisions on the use of income generated from [ACTIVITY]  **USE CODES FOR G2.03/G2.05** |
| --- | --- | --- | --- | --- | --- | --- |
| Activity code | Activity description | N106 | N107 | N108 | N109 | N110 |
| F | Non-farm economic activities: This would include things like running a small business, self-employment, buy-and-sell | YES ............ 1  NO.............. 2  🡪 ACTIVITY G | SELF………………………………...1  SPOUSE………………………....…2  OTHER HH MEMBER…………….3  OTHER NON-HH MEMBER……...4  NOT APPLICABLE……………….98  🡪 *NEXT ACTIVITY* |  | NOT AT ALL ............... 1  SMALL EXTENT ......... 2  MEDIUM EXTENT ...... 3  TO A HIGH EXTENT .. 4 |  |
| G | Wage and salary employment: This could be work that is paid for in cash or in-kind, including both agriculture and other wage work | YES .......... 1  NO ............ 2  🡪 ACTIVITY H | SELF………………………………...1  SPOUSE………………………....…2  OTHER HH MEMBER…………….3  OTHER NON-HH MEMBER……...4  NOT APPLICABLE……………….98  🡪 *NEXT ACTIVITY* |  | NOT AT ALL ............... 1  SMALL EXTENT ......... 2  MEDIUM EXTENT ...... 3  TO A HIGH EXTENT .. 4 |  |
| H | Fishing or fishpond culture | YES .......... 1  NO ............ 2  🡪 ACTIVITY I | SELF………………………………...1  SPOUSE………………………....…2  OTHER HH MEMBER…………….3  OTHER NON-HH MEMBER……...4  NOT APPLICABLE……………….98  🡪 *NEXT ACTIVITY* |  | NOT AT ALL ............... 1  SMALL EXTENT ......... 2  MEDIUM EXTENT ...... 3  TO A HIGH EXTENT .. 4 |  |
| I | Major household expenditures (such as bicycles, land, furniture, etc.) |  | SELF………………………………...1  SPOUSE………………………....…2  OTHER HH MEMBER…………….3  OTHER NON-HH MEMBER……...4  NOT APPLICABLE……………….98  🡪 *NEXT ACTIVITY* |  | NOT AT ALL ............... 1  SMALL EXTENT ......... 2  MEDIUM EXTENT ...... 3  TO A HIGH EXTENT .. 4 |  |
| J | Minor household expenditures (such as food for daily consumption or other household needs) |  | SELF………………………………...1  SPOUSE………………………....…2  OTHER HH MEMBER…………….3  OTHER NON-HH MEMBER……...4  NOT APPLICABLE……………….98  🡪 *NEXT ACTIVITY* |  | NOT AT ALL ............... 1  SMALL EXTENT ......... 2  MEDIUM EXTENT ...... 3  TO A HIGH EXTENT .. 4 |  |
| **N101/N103 DECISION CODES:**  NO INPUT OR INPUT IN FEW DECISIONS - 01; INPUT INTO SOME DECISIONS- 02; INPUT INTO MOST OR ALL DECISIONS- 03; NO DECISIONS MADE - 98 | | | | | | |

**Vegetables list and crops (900-999)**

| **Crop ID** | **Vegetable Name** | **Crop ID** | **Vegetable Name** | **Crop ID** | **Vegetable and other crops name** |
| --- | --- | --- | --- | --- | --- |
| 2 | Brinjal - Plaster | 22 | Lady Finger | 62 | Turmeric |
| 3 | Cabbage | 23 | Potato | 70 | Beans |
| 4 | Cauliflower | 27 | Spinach | 87 | Kohlrabi |
| 5 | Chili | 30 | Peas | 88 | Eggplant |
| 6 | Bottle Gourd | 32 | Coriander Seed | 89 | Arum |
| 7 | Onion | 33 | Maize | 90 | Arum-lobe |
| 8 | Green Onion | 35 | Drumstick | 93 | String Bean |
| 9 | Raddish | 38 | Carrot | 94 | Bindweed |
| 14 | Yam | 42 | Pointed Gourd | 95 | Ridged Gourd |
| 15 | Pumpkin | 43 | Cucumber | 96 | Ash-Gourd |
| 16 | Bitter Gourd | 44 | Jack Fruit | 97 | Snake Gourd |
| 17 | Pointed Gourd | 45 | Guava | 98 | Cucurbitaceous |
| 18 | Banana | 46 | Taro | 99 | Plantain (Green banana) |
| 19 | Papaya | 52 | Black Eye Beans | 900 | Wheat |
| 20 | Sponge Gourd | 54 | Lemon | 901 | Soya |
| 21 | Tomato | 56 | Gagar Lemon | 902 | Paddy |
|  |  |  |  | 903 | Jowar |
|  |  |  |  | 904 | Bajra |
|  |  |  |  | 905 | Barley |
|  |  |  |  | 906 | Maize |
|  |  |  |  | 907 | Ragi |
|  |  |  |  | 908 | Gram |
|  |  |  |  | 909 | Aarhar/Tur |
|  |  |  |  | 910 | Mung |
|  |  |  |  | 911 | Horse gram |
|  |  |  |  | 912 | Coconut |
|  |  |  |  | 913 | Cotton |
|  |  |  |  | 914 | Jute |
|  |  |  |  | 999 | Others |
|  |  |  |  |  |  |
